# Geolocation of multiple sociolinguistic markers in Buenos Aires

**DOI:** 10.1371/journal.pone.0274114

**Published:** 2022-09-09

**Authors:** Olga Kellert, Nicholas H. Matlis

**Affiliations:** 1 Department of Romance studies, Georg-August-Universität Göttingen, Göttingen, Germany; 2 Center for Free-Electron Laser Science CFEL, Deutsches Elektronen-Synchrotron DESY, Hamburg, Germany; University of Edinburgh, UNITED KINGDOM

## Abstract

Analysis of language geography is increasingly being used for studying spatial patterns of social dynamics. This trend is fueled by social media platforms such as Twitter which provide access to large amounts of natural language data combined with geolocation and user metadata enabling reconstruction of detailed spatial patterns of language use. Most studies are performed on large spatial scales associated with countries and regions, where language dynamics are often dominated by the effects of geographic and administrative borders. Extending to smaller, urban scales, however, allows visualization of spatial patterns of language use determined by social dynamics within the city, providing valuable information for a range of social topics from demographic studies to urban planning. So far, few studies have been made in this domain, due, in part, to the challenges in developing algorithms that accurately classify linguistic features. Here we extend urban-scale geographical analysis of language use beyond lexical meaning to include other sociolinguistic markers that identify language style, dialect and social groups. Some features, which have not been explored with social-media data on the urban scale, can be used to target a range of social phenomena. Our study focuses on Twitter use in Buenos Aires and our approach classifies tweets based on contrasting sets of tokens manually selected to target precise linguistic features. We perform statistical analyses of eleven categories of language use to quantify the presence of spatial patterns and the extent to which they are socially driven. We then perform the first comparative analysis assessing how the patterns and strength of social drivers vary with category. Finally, we derive plausible explanations for the patterns by comparing them with independently generated maps of geosocial context. Identifying these connections is a key aspect of the social-dynamics analysis which has so far received insufficient attention.

## Introduction

The strong impact that social circumstances have on the content and mode of people’s communication allows language to be used as a probe of human behavior and circumstances. The relationship between language and human context depends on many factors, including the identity and social role of the people involved, as well as the location and even the time of day [1]. Groups of people sharing a common characteristic, such as gender, age, ethnic or cultural identity or political orientation can also have common patterns in the style and content of their communication [2]. If these "social groups" have characteristic locations that they frequent or inhabit, spatial patterns of language use will result. Language use can also be correlated to location by the “geosocial context”, i.e., the presence of geographical and social features of a location, such as a bar, a school or a park, which influence the kinds of human activity associated with the location. Analyzing spatial patterns of language use is therefore an important tool for social sciences which provides valuable geosocial-context information. This information is applicable to a diverse range of linguistic and social issues ranging from commerce and education to geodemographics and urban planning.

Until recently, the range of possible studies has been strongly limited by the availability of data. Traditional data-taking methods, such as in-person interviews are time consuming and do not allow the kind of detailed geographical mapping or large samples of people necessary to answer some questions. Over the past decade, however, the availability of enormous amounts of social media data combining natural language and precise geolocation coordinates with user information has enabled an increasingly diverse range of socially-relevant studies that exploit the connections between language and space. Despite the intrinsic biases known to be associated with this data [3], it has been very successfully used, for example, for analysis of traffic accidents [4] and natural disasters [5], prediction of viral outbreaks [6] as well as for topic modeling [1,7] and analysis of segregation patterns in immigrant communities [8]. Social media has also provided new opportunities in geolinguistics to analyze, for example, the spatial patterns of dialect use [9–11]. These studies, however, have so far only looked at linguistic variations on large spatial scales corresponding to countries, regions and cities. On these scales, physical and administrative borders play a major role in creating language variation by restricting movement of people and also act as locations where language mixing is intensified.

Extending to smaller, urban scales, however, offers the possibility of visualizing spatial patterns of language use driven by social variables such as socioeconomic status or public context that within a city can play a larger role than borders [2,12–14]. In general, relatively few studies have been carried out in language variation on the neighborhood or urban level [1,8,13–15]. Analyzing language variations on these scales brings several challenges. The first is finding sufficient data with precise location information. Focusing on smaller and smaller scales requires increasingly large amounts of data to maintain sufficient statistics within the analysis volume. At scales of city blocks, data requirements are at the edge of what even social media sources can provide. A second challenge is that distances associated with these scales are comparable to those of daily human mobility [16], and as a result of social mixing, linguistic features tend to diffuse, causing language variations to disappear. This effect can be counteracted in cases where linguistic features are strongly connected to specific social groups or geosocial contexts that are in some way spatially segregated. Analyzing language variation on small scales therefore provides a means for studying these patterns of segregation. Mocanu et al. [15], for example, plotted the geographical distributions of different languages in the multilingual cities of Quebec and New York and showed that language polarization on neighborhood scales could indeed be resolved. Lansley & Longley [1] later used unsupervised topic-modeling to resolve spatial variations in the topics and attitudes expressed through Twitter within the city of London. A third challenge is to develop algorithms that can accurately classify categories of linguistic features. Features such as language and topic are now readily identified using machine-learning algorithms, and language classification in Twitter data is even provided as part of the tweet. Many other features exist, however, which can provide information on other social dimensions, but which have not been explored in part due to the difficulties in isolating them. The above challenges relate to the process of *measuring* spatial patterns of language use. However, once a pattern is measured, proper interpretation also requires evaluation of the statistical significance of the pattern and, more importantly, identification of the geosocial factors, i.e., *drivers* that cause the pattern. Lansley & Longley [1], addressed the latter issue by making connections between specific topics and specific regions classified by their "land use" (i.e., residential, public and parks) as well as by their function (e.g., sports stadiums and transit centers). However, not all geosocial drivers are delineated by publicly-defined administrative borders, especially on urban scales, requiring development of alternative approaches.

Our goal in this work is to develop a flexible methodology that visualizes, quantifies and explains the complex relationship between space, language and social structure. As social structure is characterized by many factors which cannot all be captured using a single linguistic feature, development of methods that can address multiple different features is needed. Our work takes steps in this direction by exploring the spatial variations in multiple aspects of language which are connected to specific geosocial contexts and social groups. We then attempt to make connections between these patterns and the corresponding city features which cause them. We identify eleven categories of language use and analyze their distributions over the greater metropolitan area of Buenos Aires using data sourced from Twitter. These categories were chosen to accomplish several goals: first, to establish baselines on the amount of variation to expect when no geosocial driver is present and when a strong geosocial driver is present; second, to extend geographical analysis to linguistic features not contained in the meaning of words but rather in the style and grammatical form, which have so far not been explored on urban scales; and third to distinguish patterns associated with geosocial context from those associated with social groups. For each case, we select contrasting sets of tweets within the same category, (also referred to as variant pairs [17–20]), and map the normalized differences in their distributions to look for patterns associated with each set. We then statistically analyze each case to evaluate the significance of the pattern and rank the different categories to determine which features provide the strongest patterns. In order to enable precise classification of tweets, we select them based on the presence of sets of “tokens”, which are linguistic elements such as words, word fragments or characters manually tailored for each category. We find distinct patterns of Twitter use for all categories where patterns are expected, except one, and derive plausible explanations based on local city features.

## Methodology

### Basic approach

The key goal of our approach is determination of the extent to which the measured patterns are driven by an underlying phenomenon connected to social groups or geosocial context. In assessing these variations, there are two aspects of especial importance: the presence of a meaningful spatial pattern, and the presence of a connection between the spatial location and the variation. To determine if the patterns are meaningful, we assess the “clarity of the pattern”, which is a measure of how clear it is that the spatial variation is not random, and look for corresponding variations in geosocial context which can plausibly explain the pattern. The pattern clarity is connected to whether variations in one location correlate to variations in other locations, and hence is related to the degree of clustering present. The second aspect will henceforth be referred to as the “signal strength”, which is a statistical measure of how strongly a particular characteristic of the location drives the variation (see section “Signal vs. noise variations” for more information on what the signal refers to). The presence of a clear pattern is often associated with a strong signal, but it need not be. These two attributes of the variation are, in principle, completely independent. In other words, it is possible to have a clear pattern without a strong signal, and vice versa. We illustrate the difference between these two concepts with an example. We consider a residential neighborhood in which the roofs of houses are either white or black. During the day, the black roofs absorb more sunlight than the white ones and consequently are warmer. The temperature of each roof is then directly related to a local characteristic (the roof color), and the resulting signal is strong. During the night, however, there is no sunlight and consequently no temperature difference, resulting in the lack of a signal. The clarity of the pattern, on the other hand, is determined by the spatial arrangement of white-roofed and black-roofed houses within the neighborhood, which is independent of the time of day. A clear pattern would result if, for example, all white-roofed houses were located north of the neighborhood and black ones to the south. Alternatively, if the houses were randomly situated, there would be no pattern, but (during the day) the signal would still be strong.

Of course, the dynamics in geolinguistics are seldom so clear cut. Connecting language use to location and the associated geosocial contexts relies on the premise that the tweeting behavior of Twitter users is strongly influenced by their local circumstances and by the social group to which they belong. Although some fraction of tweets will be completely unrelated to the local circumstance, the relevant patterns can still be observed as long as the local circumstances influence the tweets on average. This premise has already been verified for specific cases. For example, Milusheva et al. [4] showed that the most frequent locations of traffic accidents could be accurately derived based on tweets reporting on these accidents. A given “local circumstance”, however, will likely consist of a combination of influences of different strength just as a given social group likely exhibits multiple characteristics influencing their tweeting behavior in different ways. It thus remains to be determined which influences are sufficiently strong and spatially organized to produce an observable pattern. We performed our analysis on several distinct cases that highlight different categories of language use. For each language-use case, two contrasting sets of tweets, belonging to the same category, were selected and then compared. The use of contrasting sets, which we refer to as the “target” and “reference” sets, allows for a highly sensitive, differential analysis of the spatial patterns. This method of building contrastive sets that refer to the same object or concept, also called “variant pairs”, is a well-known method in dialectology [17–20]. Tweets were selected for each set by checking the text for the presence of one or more tokens which were manually chosen, based on their linguistic function, to fit a specific category. For categories defined by tokens, the tokens were chosen so that for each one in the target set there was a token of equivalent meaning and function in the reference set. This approach ensures that contributions to the tweet distributions associated with the meaning of individual tokens are the same in both distributions and thus are cancelled in the comparative analysis.

### Data selection

The data sourced from the Twitter database were filtered to only include those tweets containing precise GPS coordinates, which comprised somewhat less than 1% of the total. Using tweet data as a corpus for scientific research brings many advantages, the most important of which are the large volumes of text, the precise coordinates and the presence of metadata which can be used to track attributes of the tweets such as the language used, the user location and the user ID. These advantages come at the cost of known biases associated with the social role of the Twitter platform as well as due to large-scale automation of tweets by “bots,” incomplete or missing information about the users such as age, gender, socioeconomic status, among other things [3]. It is therefore a non-trivial question whether Twitter data or social media in general is suitable or reliable for use in language-based analyses [11].

Despite the fact that nearly 10,000 tweets are currently tweeted per second [21], data scarcity remains one of the most significant issues. As expected, the number of tweets available for analysis reduces as the size of the regions studied becomes smaller. Therefore, performing analysis on small scales places higher demands on the data than for larger scales. The same is true as the topic of study becomes narrower and more specialized. The range of topics to which this analysis can be applied is thus biased towards those with higher representation in the Corpus. To find sufficient data for urban-scale analysis, cities are therefore a natural choice. In cities, the concentration of social media users can be extremely high, providing large amounts of data even within a single city block and opening up the possibility of exploring linguistic variation on the urban scale. The concentration of social media users within a city is by no means uniform, however, necessitating care in comparing one region to another.

For our analysis, we chose the Ciudad Autónoma de Buenos Aires (CABA) because of its high density of Twitter use and because of its rich multicultural background, due to immigration in the late 18^th^ and early 19^th^ century [22,23], which creates many opportunities for linguistic variation [22,23]. Two different areas were considered in order to allow analysis of geolinguistic phenomena which occur on different scales. The first extent, which we label "CABA+," included longitudes from −58.969 to −57.844 and latitudes from −34.339 to −35.037, which encompass the provinces in the greater metropolitan area surrounding CABA in order to allow comparison of twitter use inside and outside of the city. In rough terms, this extent is used to study linguistic variations attributable to differences in characteristics between urban and sub-urban areas. The second extent, which we label "CABA," had a longitude range of −58.531506 to −58.335144 and a latitude range of −34.526514 to −34.705372 which spanned the administrative boundaries of CABA. This second extent was chosen to allow analysis of linguistic variations within the city attributable to regional variations in the geosocial properties or characteristics of the city and their associated social forces.

The time-span of the available data included October 2017 up to March 2021, and for all studies but one, the tweets were further filtered to include only those written in Spanish (i.e., Lang = ‘es’). The result was a set of 1,065,614 tweets for CABA and a set of 1,930,987 tweets for CABA+, which we refer to as the *Corpus*. For the purposes of comparison, two cases also used tweets written in English (i.e., Lang = ‘en’) in CABA and CABA+. A full set of Tweet IDs can be found in S1 and S2 Files. Data were collected by using the Twitter Application Programming Interface (API), in accordance with Twitter’s Developer Agreement & Policy. We do not display or republish any Twitter content to comply with the Twitter terms of service.

### Binning and visualization on maps

The basis of our computational approach is to calculate and compare the spatial distributions of target and reference tweets within the chosen extent. To calculate the distribution, we define a 2-dimensional grid of bins distributed over the spatial extent of the city and count the number of tweets from each of the two sets belonging to each bin. For CABA+, the extent was divided into 100 x 100 bins, resulting in bins with a size of about 1 km. For CABA, 75 x 75 bins were used, resulting in bins slightly larger than 200 m. For reference, city blocks within CABA have dimensions of order 100 m by 100 m. The binning therefore allowed resolution of features on the scale of city blocks, neighborhoods and regions within the city corresponding to various functionalities, such as business and residence. To allow a precise description of the visualized patterns, we define several quantities. For every bin in the grid, we first count the number of all tweets, $c_{i,j}^{A}$, whose GPS coordinates lie within the boundaries of the bin in the *i*^*th*^ column and *j*^*th*^ row of the grid as well as the counts $c_{i,j}^{T}$ and $c_{i,j}^{R}$ of the target and reference tweets within each bin, respectively. To visualize the distributions, markers representing the tweet counts from each bin were overlaid onto a geographical map using the Python package Cartopy [24].

**Fig 1:**
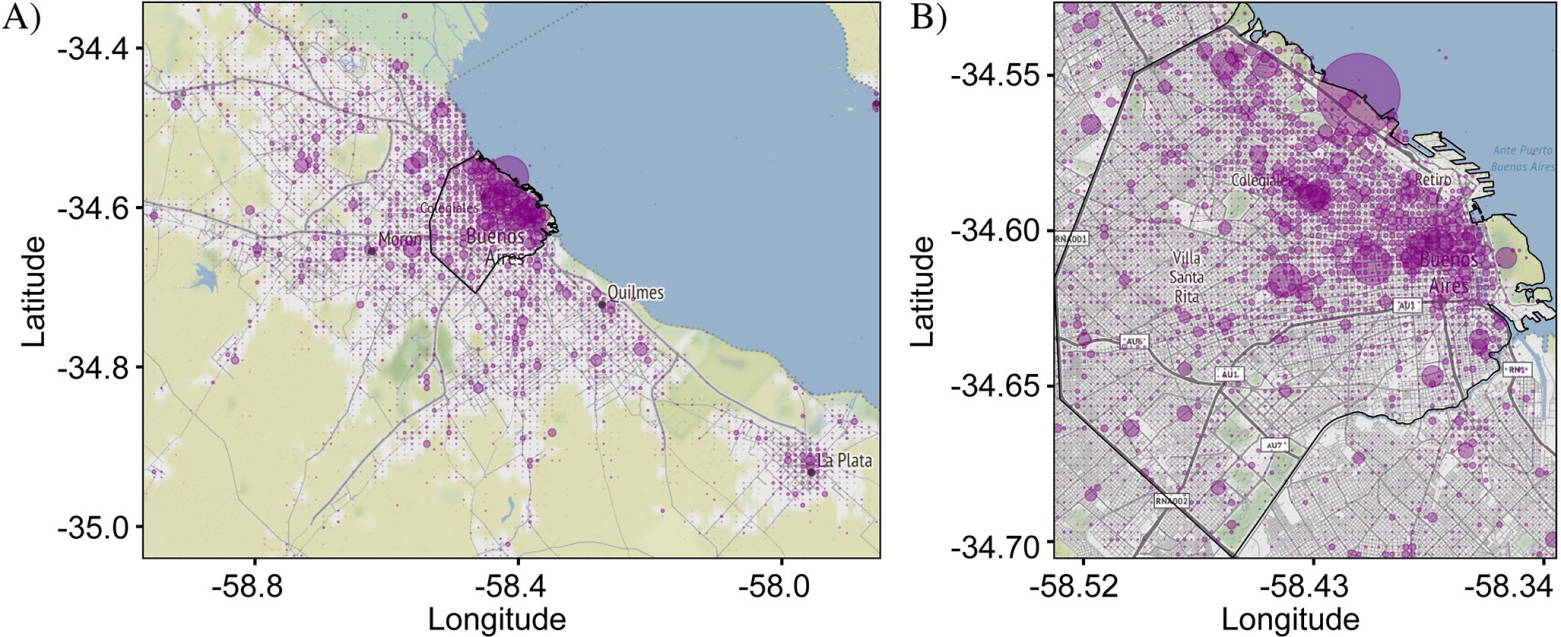
Geographical plot of the spatial distribution of all Spanish tweets in Buenos Aires. Each circle corresponds to an individual bin and its size represents the number of tweets in that bin. (A) Distribution in the greater metropolitan area of Buenos Aires (CABA+) which is sub-divided into 100 x 100 bins. (B) Distribution in the administrative boundary of the city of Buenos Aires (CABA)–delineated in black–which is sub-divided into 75 x 75 bins. Both distributions show greater tweet counts in the more population-dense regions corresponding to the downtown area (upper-right quadrant of (A)). The total number of tweets in the CABA+ and CABA extents were 1,930,987 and 1,065,614, respectively. Base map and data from OpenStreetMap and OpenStreetMap Foundation under the Open Database License.

Fig 1 shows plots of $c_{i,j}^{A}$ representing the distribution of all Spanish tweets in CABA+ and CABA from our Corpus. A circular marker represents the center of each bin and the number of tweets is represented by the size of the markers which are semi-transparent to avoid obscuration of neighboring markers. Overlapping of multiple markers deepens the color, helping to emphasize locations with large tweet densities. Inspection of the distribution shows that the tweet density tracks the regions of development of the city and hence the population density. Specifically, the tweet density is highest in the city center and follows the major roadways. This general pattern is to be expected for all tweet distributions, since, all other things being equal, more people create more tweets. As a result, distinguishing variations between target and reference distributions can be challenging, especially when the variations are subtle.

**Fig 2:**
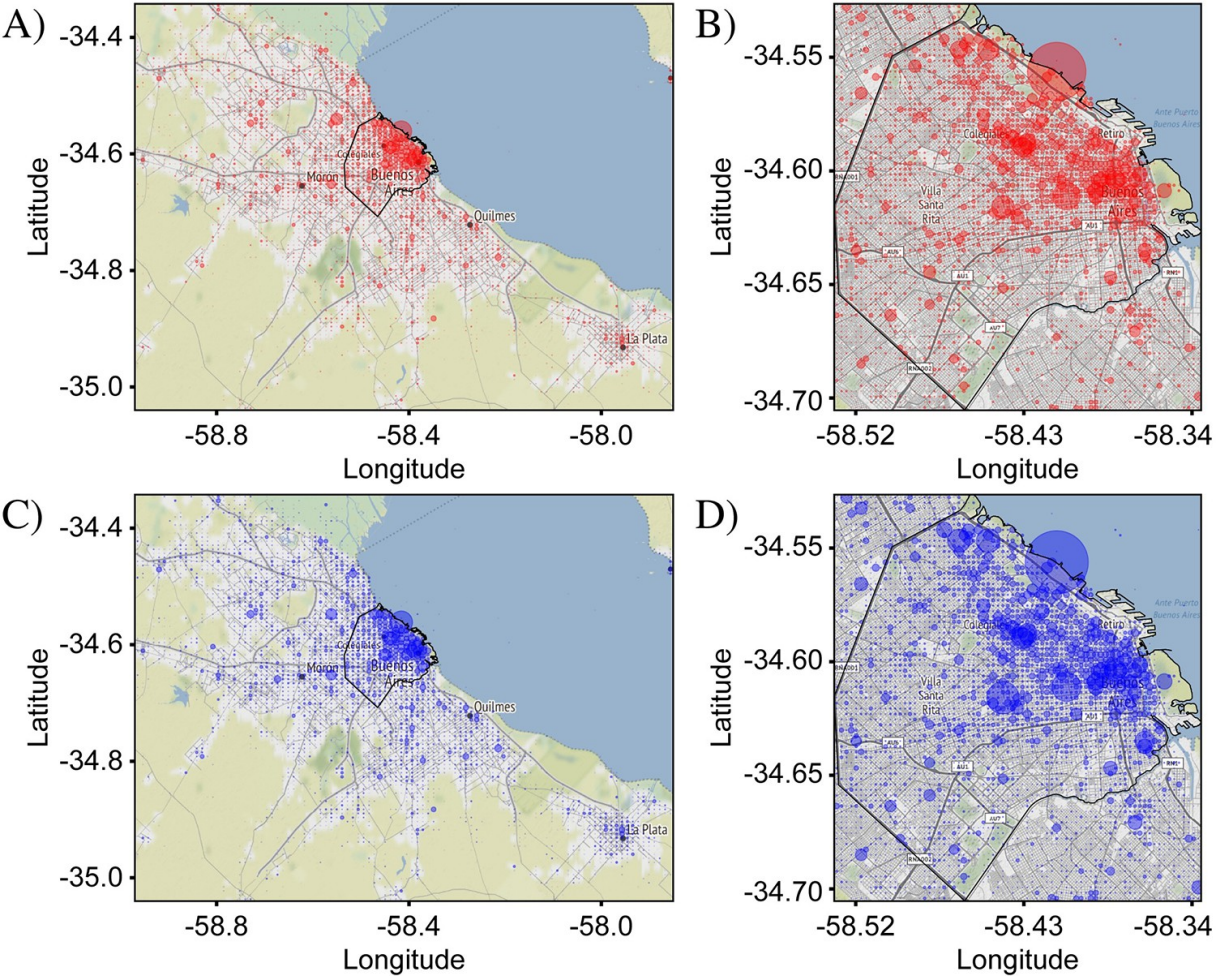
Comparison between distributions of tweets containing the letter ‘v’ and ‘b’ in CABA+ and CABA. Letter ‘v’ is represented in A and B by red circles. Letter ‘b’ is represented in C and D by blue circles, respectively. The size of each circle represents the number of tweets in the corresponding bin for each case. As ‘v’ and ‘b’ are orthographic elements, without independent meaning, no difference in their distributions is expected. In fact, the measured distributions are nearly identical to each other, with no visually noticeable differences. The distributions are also nearly identical to the distribution in Fig 1 showing all Spanish tweets, indicating that the dominant pattern is associated with the population density: The highest density of tweets is in the downtown area, while significantly lower densities are in the surrounding areas. Both points illustrate the need for a representation that highlights small differences in the distributions to enable a sensitive analysis. The total number of ‘v’ and ‘b’ tweets were 325,514 and 394,175, respectively. Base map and data from OpenStreetMap and OpenStreetMap Foundation under the Open Database License.

In order to illustrate this point, we compare the distributions of tweets containing the letters ‘v’ and ‘b’. As these letters are orthographic elements which do not carry lexical meaning by themselves, no major spatial variations are expected in the use of one versus the other. Small variations, however, due to noise will exist in every measurement. This comparison therefore offers an ideal way to test the sensitivity of our method to detect the variations. In Fig 2, the distributions of tweets with the two letters are plotted over both the CABA+ and CABA extents. As predicted, the two distributions appear nearly identical to each other as well as to the distribution of all tweets in Fig 1. Small differences are not easily discernable by visual inspection. To see the differences requires a representation, i.e., a metric, which emphasizes locations where the distributions deviate from each other.

A typical approach when comparing distributions of variant pairs is to calculate the proportions, i.e., the "relative frequencies," of each variant [25,26]. This metric, which for our bins correspond to the values $c_{i,j}^{T}/{(c}_{i,j}^{T}+c_{i,j}^{R})$ and $c_{i,j}^{R}/{(c}_{i,j}^{T}+c_{i,j}^{R})$ for the target and reference tweets, respectively, has recently been used for analyzing geographic variations in language use [13,15,19]. This metric, however, has a number of undesirable features for our analysis. First, it tends to give equal weight to all bins, independent of count numbers, so that in bins only containing tweets of a single variant, the proportion of this variant will be 100% whether there is one tweet or a thousand. Second, the metric is undefined for bins with no tweets. And third, the value of the relative frequency tends to reflect the ratio of the total number of target vs the total number of reference tweets and thus does not vary much in value for small bin-to-bin variations in tweet counts. While we found the relative frequency metric to be good for revealing spatial patterns in the use of each variant when the regions associated with each were clearly demarcated, it was less sensitive when the variations were subtle and spatially intermixed. In addition, we found the equal weighting to provide a misleading representation of the significance of the variations by over emphasizing the importance of regions with low tweet counts. Finally, for cases where the overall number of tweets of each variant differed by a large amount, small variations in the relative frequencies were problematic to visualize.

Here we describe an alternative metric based on the normalized difference in target and reference tweet counts per bin and show how this metric addresses the above issues. The mathematical formulation is given below:

We first define normalized tweet distributions by: $f_{i,j}^{T}\equiv c_{i,j}^{T}/N^{T}$ and $f_{i,j}^{R}\equiv c_{i,j}^{R}/N^{R}$, where $N^{T}\equiv \sum_{i}\sum_{j}c_{i,j}^{T}$ and $N^{R}\equiv \sum_{i}\sum_{j}c_{i,j}^{R}$ are the total number of tweets in the target and reference distributions respectively. The quantities $f_{i,j}^{T}$ and $f_{i,j}^{R}$ represent the fraction of tweets in the (i,j)^th^ bin for the target and reference cases, respectively. The comparison between the two distributions is then done by calculating the difference in the tweet fraction per bin: $\Delta f_{i,j}\equiv f_{i,j}^{T}-f_{i,j}^{R}$, which henceforth we refer to as the “differential distribution”. This quantity can be interpreted as follows: bins with positive values of Δ*f*_*i*,*j*_ over represent the target tweets while negative values under represent them, relative to the reference-tweet distribution. Since bins with equal representation of tweets have Δ*f*_*i*,*j*_ = 0, independently of the total numbers of each variant, small variations in degree of representation can sensitively be resolved. This metric does not require special treatment for bins with zero counts, and results in larger values of Δ*f*_*i*,*j*_ for larger variations, even if either of the tweet counts are zero. As a result, noise associated with low-count bins is suppressed. A consequence of the normalization is that the sum of the distribution differences is exactly zero: ∑_*i*_∑_*j*_Δ*f*_*i*,*j*_ = 0, so that for any two distributions, the contributions from each will be equal and that all bins will be identically zero (Δ*f*_*i*,*j*_ = 0) for two distributions of exactly the same shape but a different total number of tweets.

**Fig 3:**
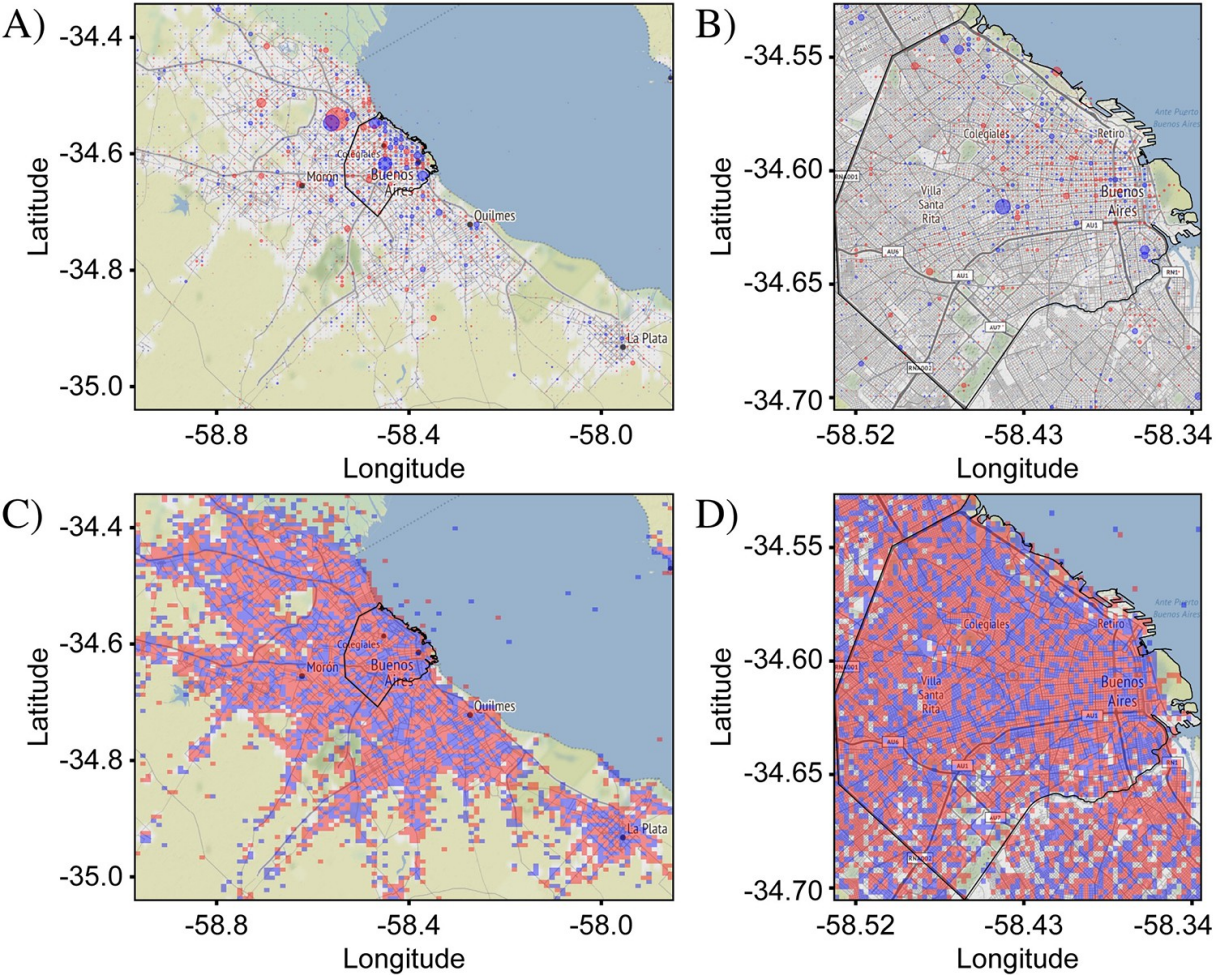
Differential distribution of ‘v’ and ‘b’. The differential distributions represent the difference between fractional counts of target and reference tweets (here, tweets containing the letter ‘v’ and tweets containing the letter ‘b’, respectively) in each bin. Positive values, plotted in red, show where target tweets are more prominent relative to the average, while negative values, plotted in blue, show where the reference tweets are more prominent. Two representations are shown. In (A) and (B), circles centered at each bin have a size proportional to the value of the fractional difference, thus capturing the magnitude of the differential variations in target and reference tweet densities. In (C) and (D), the square area of each bin is uniformly filled with red or blue, capturing only the sign of the fractional difference which helps to visually identify spatial patterns in the difference between the target and reference distributions. Both representations allow sensitive visualization of small differences not visible in Fig 2. (A) and (C) were plotted over the CABA+ extent, while (B) and (D) were plotted over the CABA extent. The black outline represents the administrative borders of CABA. In both representations and both extents, the differential distributions appear to have no significant pattern, consistent with expectation. Tweet counts: For CABA+, N^T^ = 560,334 and N^R^ = 657,233. For CABA, N^T^ = 325,514 and N^R^ = 394,175. Base map and data from OpenStreetMap and OpenStreetMap Foundation under the Open Database License.

Fig 3 confirms that the *differential distribution* approach enables a highly sensitive analysis of spatial variations. The differential distributions shown were calculated from the data in Fig 2. Two different visualizations are presented which highlight different aspects of the variations between the target and reference tweet distributions. In the first visualization (Fig 3A & 3B), the size of a circular marker is used to represent the magnitude of Δ*f*_*i*,*j*_ and the marker color was used to represent the sign of Δ*f*_*i*,*j*_, with red and blue for positive and negative values of Δ*f*_*i*,*j*_, respectively. This (“circle”) visualization emphasizes bins where the magnitude of Δ*f*_*i*,*j*_ is large and de-emphasizes bins where it is small, allowing easier interpretation of the most prominent variations.

In the second visualization (Fig 3C & 3D), square markers with dimensions matching the dimensions of the bin were used and once again red and blue colors were used to represent the sign of Δ*f*_*i*,*j*_. As a result, the bins were effectively colored according to the relative prominence of the target tweets. This (“square”) visualization de-emphasizes the magnitude of the variations, as the colors are assigned in a binary fashion, and thus captures the benefit of the relative fraction approach in allowing an easier interpretation of the spatial *patterns* associated with each distribution. In both visualizations, locations where the differential distributions vary are now highlighted, but as can be seen in the example of Fig 3, the patterns are highly complex and thus require robust methods of evaluation.

### Quantification and evaluation of the variations

As introduced in the section “Basic Approach”, evaluation of a language-use case requires assessment of the strength of the signal, the clarity of the spatial pattern, and the similarity of the pattern to the spatial distribution of an identifiable characteristic of the city. Our evaluation combines a number of objective and subjective analyses for each of the cases we study.

To quantify the signal strength, we assess the degree to which the number of target tweets in each bin is correlated to the number of reference tweets. This analysis is based on the assumption that, in the absence of a driver of variation, the number of tweets of any category will primarily be determined by the population density [27]. As a result, the relative likelihood of target and reference tweets will be unaffected by position, and therefore, the ratio of target to reference tweets in any particular bin will be the same as the ratio of target to reference tweets in the corpus. In other words, the number of target tweets ($c_{i,j}^{T}$) will be linearly related to the number of reference tweets ($c_{i,j}^{R}$), with a proportionality constant determined by the numbers of each tweet variant in the corpus ($c_{i,j}^{T}=kc_{i,j}^{R}$, where *k* ≡ *N*^*T*^/*N*^*R*^). The effect of any spatially-dependent driver of variation is to cause certain locations to favor target tweets and others to favor reference tweets. Necessarily, the relationship between the target and reference tweet counts will then deviate from linear. To quantify this deviation, we perform a linear regression and calculate the Pearson correlation coefficient (*r*_*PCC*_) [28,29], which is a direct measure of how far the data deviates from linear. A perfect correlation (equal distributions) results in a Pearson coefficient of *r*_*PCC*_ = 1, while completely uncorrelated data (no similarity between the distributions) yields a coefficient of *r*_*PCC*_ = 0. Since the inverse relationship between correlation coefficient and signal strength is somewhat counter intuitive, we define the signal strength as one minus the Pearson correlation coefficient: *S*_*PCC*_ ≡ 1−*r*_*PCC*_. With this definition, the signal strength is zero for perfectly correlated data and one for uncorrelated or anti-correlated data.

Determining this degree of deviation is related to the problem of determining the probability that the target and reference tweet data sets come from the same spatial distribution. The general problem of comparing two distributions is often addressed by performing the 2-sample Kolmogorov-Smirnov (K-S) test which evaluates the maximum difference in the cumulative probability distribution [30]. For comparison, we therefore compute the Kolmogorov-Smirnov statistic (KSS) for each of our cases.

It should be noted that our PCC-based metric does not incorporate any spatial information and thus does not evaluate the presence of a spatial pattern. It only quantifies the degree to which a variation is present. Assessment of the presence or clarity of the pattern is therefore done in a separate step. We first evaluate the pattern subjectively by visual inspection of the differential distributions and look for features that appear relevant based on the geography. For each case, we search for plausible explanations for the patterns by examining the spatial distributions of relevant social variables and checking for correspondence with the patterns in the differential distribution. To provide a quantitative assessment of the pattern clarity, several tools exist. Among the most important of these is Moran’s *I* statistic, which evaluates the degree of clustering in the pattern by performing a spatial autocorrelation and is frequently used to assess the statistical significance of patterns [11,31]. The autocorrelation statistics comes in global and local variations. The *global* Moran’s *I* statistic (GMI) is a single value that measures the overall degree of clustering in the whole image, while local statistics (such as Getis-Ord Gi* [19,31]) enable a localized assessment of clustering significance. For our analysis we calculate the GMI for each differential distribution and use a weighting matrix that includes only nearest neighbors, as is frequently done. Although the local metric can provide more information and is clearly pertinent for interpreting relevant features of a pattern, our main purpose is to investigate a new approach and new directions for further study in urban-scale geolinguistic analysis. It is beyond the scope of this work to make a complete or definitive analysis of any particular differential distribution, so we leave the local analysis for more detailed follow-up of particular cases. A comparison of the results of these statistics is done at the end of the manuscript, in section “Statistical comparison of all cases”.

### Visualization of the signal strength

**Fig 4:**
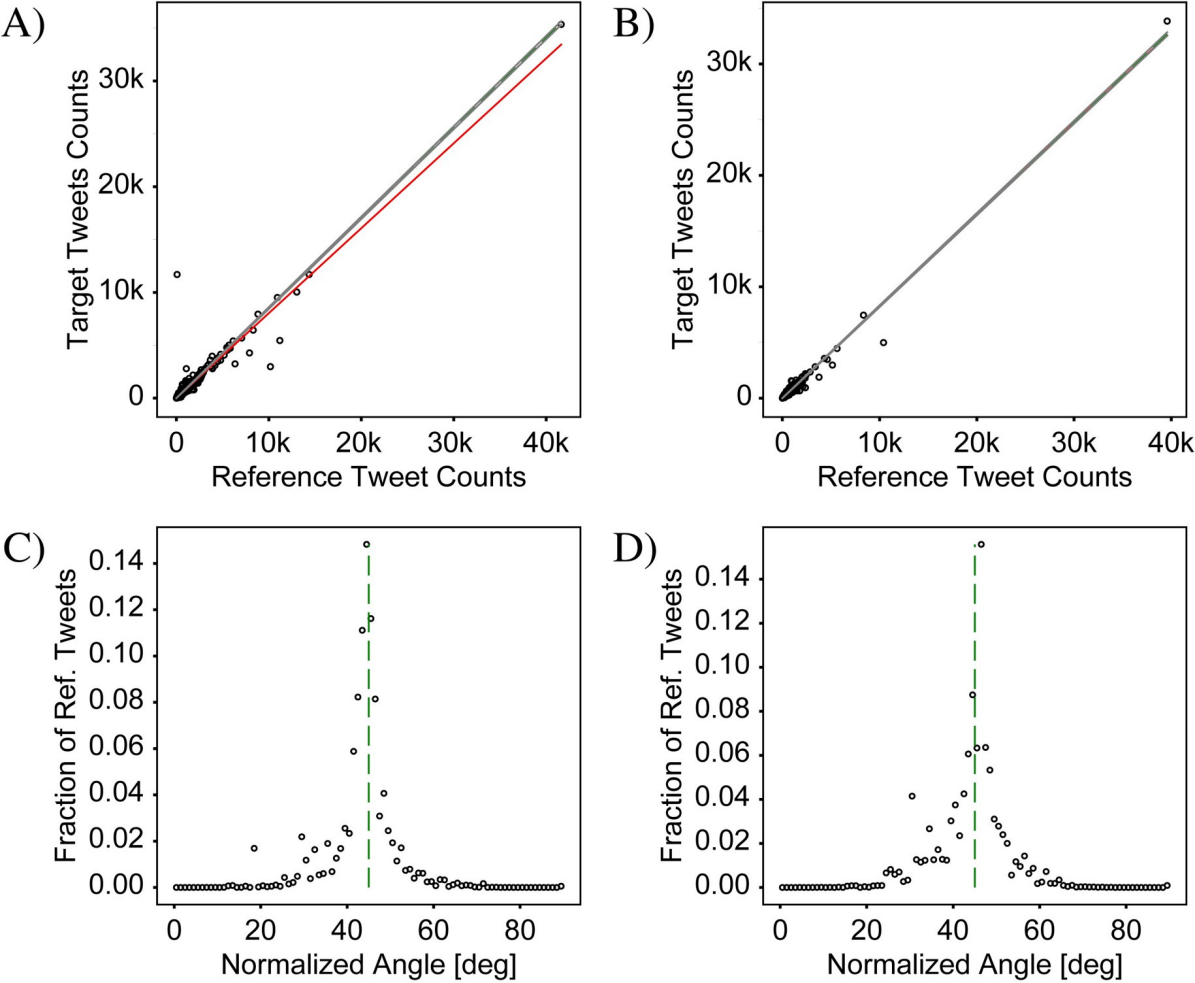
Correlation analysis for the data in Fig 3. (A) & (B) “Frequency comparison plots” in which target tweet counts are plotted against reference tweet counts for each bin for the CABA+ and CABA extents, respectively. Both plots show a high degree of correlation between target and reference tweet counts, indicating the lack of a driver of variation. A green-dashed line is used to represent the case of exact correlation, while the red line represents the best-fit line resulting from a regression analysis. Surrounding the exact-correlation line are two gray lines indicating the range of expected deviation resulting from random statistical variation when counting discrete elements (i.e., shot noise). (C) & (D) Plots of the normalized angle histograms corresponding to (A) and (B), respectively. The angle corresponds to the angle of a line drawn from the origin of the frequency comparison plot to an individual point after normalizing the counts by their respective sum. These histograms provide a complementary assessment of the degree of correlation between the target and reference tweet counts by showing how tightly the points are clustered around the exact-correlation line (again indicated by a green-dashed line). The two analyses differ in that the regression is more strongly affected by bins with high tweet counts while the histogram weighs all bins equally. The analysis here shows that counts of ‘v’ tweets and counts of ‘b’ tweets are highly correlated confirming lack of a driver of variation. CABA+ Statistics: N^T^ = 560,334, N^R^ = 657,233, S_PCC_ = 0.04, KSS = 0.02, GMI = 0.01. CABA Statistics: N^T^ = 325,514, N^R^ = 394,175, S_PCC_ = 0.01, KSS = 0.01, GMI = 0.03.

To aid in visual interpretation of the signal strength, we plot two types of graphs. The first, which we henceforth refer to as a “frequency-comparison plot”, is a scatter plot which plots all the bins using ($c_{i,j}^{R},\;c_{i,j}^{T}$) as the x,y coordinates (Fig 4A & 4B). As a reference for the exact-correlation condition, the line defined by $c_{i,j}^{T}=kc_{i,j}^{R}$ is displayed on each scatter plot as a green dashed line. The extent to which the points do not lie on the “exact-correlation” line indicates how different the two distributions are. Points above and below the line correspond to areas that over- and under-represent target tweets, respectively, relative to the reference tweets, and are indicated by red and blue markers in the differential-distribution plots, respectively. The best-fit line from this regression analysis is also displayed on each plot as a solid red line. A discrepancy between the slopes of the best-fit and exact-correlation lines shows that the ratio between target and reference tweet counts varies between bins with low and high tweet counts. It should be noted that the data in the *frequency-comparison* plots is inherently positively skewed, as negative count values are not possible and because, in general, there are fewer bins with high tweet counts than with low tweet counts. As a result, bins with large tweet counts can disproportionately affect the value of the correlation coefficient compared to the low-count bins. Nevertheless, we find that the signal strength based on the Pearson correlation coefficient provides a metric that matches well with expectations based on visual inspection of the frequency-comparison plots. A more detailed discussion on interpreting these plots and inferring properties of the geosocial driver is provided in the last section, entitled “Statistical comparison of all cases”.

The second type of graph is designed to provide an easier assessment of the amount of spread away from the *exact-correlation* line of the points in the frequency-comparison plots. Since each frequency-comparison plot contains data from several thousand bins, the points overlap and become undistinguishable, making it difficult to determine how the density of points varies, especially in cases with a high degree of correlation. To address this issue, we bin the points according to the degree of deviation from the exact-correlation line. To capture an intuitive sense for the deviation, we define an angular metric, calculated using $\theta_{i,j}\equiv \tan^{-1}\;c_{i,j}^{T}/c_{i,j}^{R}$, which is the slope of the line connecting each point with the origin. A histogram of the angles is then created with bins of width one degree, over the range from 0–90 degrees (Fig 4C & 4D). For exactly correlated data, all the points would have the same angle corresponding to the slope of the *exact-correlation* line, and the histogram would show a peak with zero width at this angle. For increasingly non-correlated data, the width of this peak grows from zero to a finite value which characterizes the degree of non-correlation. Completely non-correlated data would no longer show a peak, but rather would have an angle histogram which is completely flat. The benefit of this metric is that it allows for a measurement of the ratio of the target and reference tweet counts, but gracefully handles the cases where either $c_{i,j}^{T}$ or $c_{i,j}^{R}$ is equal to zero. In contrast to the frequency-comparison plot, where bins with larger values of $c_{i,j}^{T}$ and $c_{i,j}^{R}$ have a relatively larger impact on the best fit and correlation coefficient, the calculated angles do not preserve information about the count numbers. As a result, the angle histogram gives equal weight to bins of high and low count numbers, and is thus helpful for addressing sample sets with large positive skew. When the total numbers of target and reference tweets are very dissimilar resulting in exact-correlation lines with angles near 0 or 90 degrees, however, the width of the histogram peak is no longer comparable to the width in the case of well-balanced data. This effect is due to the nonlinear nature of the inverse-tangent function used to calculate the angle. We therefore consider instead the normalized angles defined by $\theta_{i,j}^{N}\equiv \tan^{-1}\;f_{i,j}^{T}/f_{i,j}^{R}$, which results in a histogram centered around an exact-correlation line with an angle of 45 degrees, making the widths of the histograms more comparable to each other.

### Signal vs. noise variations

In order to evaluate the observed patterns, it is important to understand what sources can contribute to them. We divide these sources broadly into two categories: “signal” and “noise”. By *signal*, we mean a pattern of variation that can be connected to or explained by a geosocial factor, resulting in a meaningful pattern. *Noise*, on the other hand, refers to sources of variation that do not correlate to the particular phenomenon being studied and thus creates a background which obscures the signal. Noise sources can include many factors, including: 1) random statistical variations arising in any measurement of discrete quantities, i.e., “shot noise” [32,33] such as in the classic coin-toss experiment [34]; 2) inherent variability of human behavior, such as making mistakes or changing one’s mind; 3) variations due to unanticipated or unresolvable correlations with location; 4) skewing of patterns due to highly automated tweeters (i.e., “bots”); and 5) systematic errors in the data. The first category of noise can be estimated by assuming it results from a random process. In random processes, the noise level associated with counting *N* individual events is equal to $\sqrt{N}$. The statistical noise in the target distribution can thus be described by $\Delta c_{i,j}^{T,stat}\equiv k\sqrt{c_{i,j}^{R}}$. To display the calculated level of shot noise expected from this source, we added grey lines to the frequency comparison plots on either side of the exact-correlation line and separated from the line by an amount $\pm \Delta c_{i,j}^{T,stat}$. The second and third categories of noise include associations that are not visible to an external observer between specific text in the tweets and specific locations. These associations can come from any number of complex factors. The fourth category of noise, “bots”, refers to Twitter accounts which generate tweets in an automated fashion. The automation allows relatively few users to generate tweets in extreme quantities. It has been estimated that as many as 24% of tweets come from bots [35]. Identifying bots and evaluating their influence is therefore clearly important, but finding distinguishing features is not trivial. Noise in the last category can arise due to inaccuracies in the geolocation data. Although GPS data can in principle provide positioning information on scales down to a meter or less, the accuracy of the geolocation data stored in the tweets can depend on the device used, and so is not guaranteed [36]. Determining the degree of accuracy is not straightforward, as there are no direct references to use for comparison. However, based on a preliminary analysis showing evidence of inaccuracies in geolocation of tweets with GPS coordinates containing 4 digits of precision or fewer, we included only those containing 5 or more digits.

## Analysis of geolocation patterns

The goals of our analysis are twofold: to demonstrate that spatial patterns connected to various types of linguistic features can be measured on the urban scale and to show that these patterns are meaningful. In order to show they are meaningful, it is necessary to estimate the levels of variation that can be expected from noise sources, as described above. Our first study is therefore of “null” cases where variations are not expected, in order to provide a baseline on the level of non-meaningful variations to expect in any pattern. In order to target the meaningful cases, we study several cases where we expect to find robust patterns that are relatively easy to interpret. We break these cases into two categories. The first category is those where the pattern can be attributed to geosocial context, while the second category is connected to social groups.

### Variation due to noise sources

**Fig 5:**
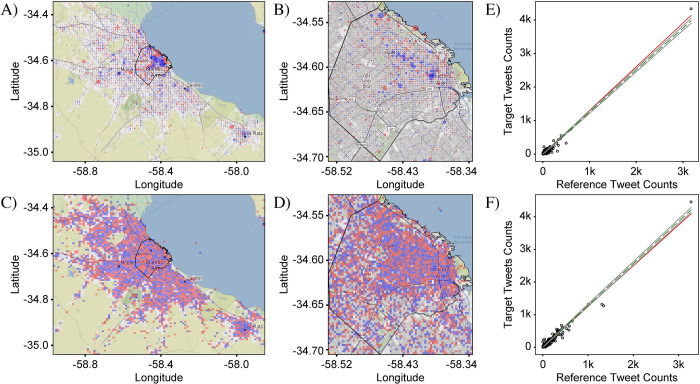
Null case “las” vs “los”. (A) & (B) are the differential distributions of plural determiners in Spanish “las” (feminine) vs. “los” (masculine) plotted over the extents of CABA+ and CABA, repectively, using the “circles” representation that highlights magnitudes of differences. Blue (red) shows where “los” (“las”) is more prominently used. (C) & (D) are the same as (A) & (B), except plotted using the “squares” representation which highlights spatial patterns. All four graphs show that “los” and “las” are just about equally represented across the city. (E) & (F) are the “frequency comparison plots” for CABA+ and CABA respectively, and show that counts of “las” correlate very well with counts of “los”, indicating the lack of a strong variation signal on either scale. CABA+ Statistics: N^T^ = 65,573, N^R^ = 51,033, S_PCC_ = 0.02, KSS = 0.02, GMI = 0.15. CABA Statistics: N^T^ = 38,870, N^R^ = 31,021, S_PCC_ = 0.02, KSS = 0.02, GMI = 0.09. Base map and data from OpenStreetMap and OpenStreetMap Foundation under the Open Database License (Fig 5A–5D).

We first study two “*null*” cases which are those for which we expect *no* significant difference in the shape of the target and reference distributions (Fig 5). For these cases, we therefore selected sets of target and reference tokens that do not express different meanings by themselves, but have the same or similar grammatical or social function. These sets are then expected to be used in similar locations since they do not directly or indirectly mark a location.

For the first null case, we use the comparison of tweets containing the letters ‘v’ and ‘b’ presented in Figs 2–4. As mentioned above, since these letters have no independent meaning and are phonetically similar, no prevalence for either is expected in any location. This conclusion is confirmed by Fig 3 which shows that the magnitudes of the variations are small for both CABA+ and CABA extents. The red and blue points are evenly distributed over the map, with no obvious spatial pattern, although a few larger variations tend to occur in the downtown area of CABA (upper-right quadrant of the graph). The larger variations downtown are expected due to the larger concentrations of people and hence larger overall density of tweets, as seen in Fig 1.

The frequency-comparison plots in Fig 4A & 4B show that the counts of target and reference tweets in each bin are strongly correlated, with the points clustered tightly around the best-fit line. The linear regression analysis yields a Pearson coefficient near one of 0.96 and 0.99 for CABA+ and CABA, respectively. The corresponding plots in Fig 4C & 4D confirm a strong clustering of the angles around the 45-degree mark associated with perfect correlation. Nevertheless, deviations of the points in the scatter plot from the exact-correlation line are noticeable and give us a measure of the level of variation to be expected from noise sources. Specifically, Fig 4A & 4B show that many of the points lie outside of the envelope associated with shot noise, delineated by the two grey lines on either side of the green exact-correlation line. Since the condition for including tweets is not very exclusive, the tweet counts for this case are very high. As a result, the level of shot noise is low relative to the counts, as can be seen by the proximity of the gray lines to the green-dashed line in frequency-comparison plots. Despite the high level of correlation in this data, the observed variations for many of the points are multiple times larger than can be explained by pure statistical noise. These variations are most likely due to contributions from other noise categories. A likely candidate is accidental spatial associations of particular letters to particular locations, such as ‘v’ in the airport because of the connection to ‘vacation’ or ‘b’ in locations with names containing this letter (e.g., ‘La Boca’).

The second null case compares tweet distributions for the target set {*las*} and reference set {*los*} (Fig 5). The tokens *las* and *los* are the plural-feminine and plural-masculine versions of the definite article, ‘the’, and as they have the same linguistic function and are not socially marked, no strong variation is expected. The differential distribution for both extents and in both representations are shown in Fig 5A–5D. As with ‘v’ vs ‘b’, the definite articles show no strong spatial variations in their distribution. Similarly, the frequency-comparison plots (Fig 5E & 5F) show a very strong correlation, indicating the lack of a variation signal.

### Signal variations from language use connected to geosocial context

We next examine several cases of increasing complexity in which language-use is connected in different ways to geosocial context. Geosocial context, here, is defined broadly to capture any aspect of the location that can influence tweeting behavior. The first two cases examine tweets containing names of two specific locations. Since a direct and unique link exists between each name and the corresponding location, a very strong pattern of variation is expected. The third case considers tweets containing names of two activities. In this case, the link between the tokens and the locations is still direct [1,5], but because the activities can be associated with multiple locations of the same type, such as the venues in which they can be performed, the patterns are more complex. Lastly, we compare the distribution of tweets of two different language styles. In this case, there is no direct connection between the tokens and the locations. Instead, the link is between the type of language and the type of location, which necessarily is more subtle. The progression of cases serves to illustrate different aspects of geosocial context which have influences of varying strength.

**Fig 6:**
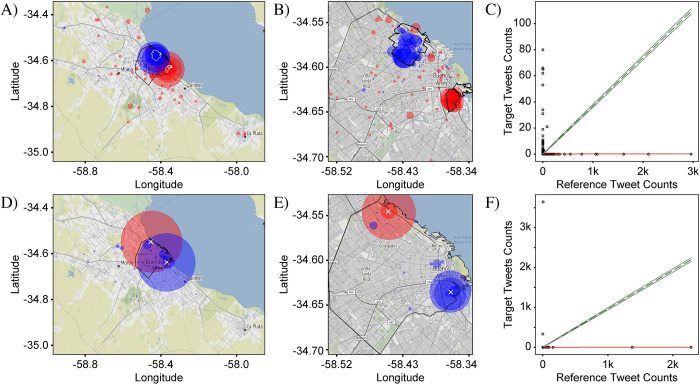
Neighborhood names and location names cases. (A) & (B) Differential distribution of neighborhood names “La Boca” and “Palermo” plotted over the extents of CABA+ and CABA, respectively. Blue (red) circles show where “Palermo” (“La Boca”) is more prominently used. The distributions show that tweets mentioning the neighborhood names are highly localized to the neighborhoods themselves which in (A) are delineated by white outlines and in (B) by black outlines. These figures also show that of the two neighborhoods, La Boca is more likely to be mentioned by tweeters outside the confines of the two. (C) “Frequency-comparison plot” for the data in (B) showing that the counts of tweets mentioning “Palermo” almost perfectly anticorrelate to counts of tweets mentioning “La Boca”. This anticorrelation indicates a very strong spatial influence on the tweeting, with few bins having tweets mentioning both neighborhoods. CABA+ Statistics N^T^ = 632, N^R^ = 16,062, S_PCC_ = 0.998, KSS = 0.87, GMI = 1.07. For CABA Statistics: N^T^ = 590, N^R^ = 15,967, S_PCC_ = 0.999, KSS = 0.89, GMI = 0.70. (D) & (E) Differential distribution of the two most important soccer stadium names “La Bombonera” and “‘Monumental’ Antonio Liberti V” in CABA+ and CABA, respectively. Blue (red) circles show where “La Bombonera” (“‘Monumental’ Antonio Liberti V”) is more prominently tweeted. As with neighborhood names, tweets mentioning the stadiums almost exclusively come from the location of the stadiums themselves (marked by white crosses), indicating that being in the presence of the stadium is a strong driver for tweeting about it. In particular, “‘Monumental’ Antonio Liberti V” is only mentioned in two bins, both of which are in the immediate vicinity (or even within) the stadium. “La Bombonera” is mentioned slightly more extensively, but not dramatically so. (F) “Frequency-comparison plot” for the data in (E) showing that the counts of tweets mentioning the two stadiums almost perfectly anticorrelate to each other. CABA+ Statistics: N^T^ = 3,978, N^R^ = 744,539, S_PCC_ = 0.999, KSS = 0.998, GMI = 0.06. CABA Statistics: N^T^ = 3,978, N^R^ = 4,131, S_PCC_ = 0.999, KSS = 0.999, GMI = 0.65. Base map and data from OpenStreetMap and OpenStreetMap Foundation under the Open Database License (Fig 6A, 6B, 6D and 6E).

For the first case, the target and reference tokens selected were the names of two important neighborhoods in CABA, “La Boca” and “Palermo”, respectively (Fig 6). As shown in Fig 6A & 6B (for CABA+ and CABA extents, respectively), the difference in the distributions is striking. Tweets mentioning each of the names are highly localized to the respective neighborhoods (the geographical borders of which have been marked on the graphs) and have very little geographic overlap with each other. The corresponding frequency-comparison plot in Fig 6C shows that target and reference tweet counts almost perfectly anti-correlate, confirming that the two distributions are nearly entirely distinct. Together, the presence of a clear pattern and a strong variation signal reinforce the conclusion that physical presence in the neighborhood strongly influences the likelihood of mentioning the neighborhood in a tweet. It should be noted, however, that outside of the two neighborhoods, La Boca is more likely to be mentioned than Palermo, as shown by the prevalence of red circles throughout both the CABA+ and CABA extents. This asymmetry may indicate something about the relative importance of the two. It is also true that the number of tweets mentioning La Boca is much larger than the number mentioning Palermo, by about 30 to one. This asymmetry in tweet counts, however, does not affect the conclusions above, as we show in the supplementary information (Figs 1–3 in S5 File).

For the second case, the search words were the names of two soccer stadiums: *Estadio La Bombonera* and *Estadio Monumental Antonio Liberti V*. Here, the separation between the two distributions (Fig 6D & 6E) is even more extreme, and the frequency-comparison plot (Fig 6F) shows a perfect anti-correlation. The two geographical plots do not require additional analysis to see that the pattern is “meaningful” and is driven by the presence of the user in each of the locations (marked by x’s in the graphs). The high level of anti-correlation in the frequency plots confirms that there is also a very strong signal. The GMI value for the CABA+ extent is surprisingly low, given the extreme clarity of the patterns. As discussed in the section “Comparison between statistical metrics” and in the discussion, this result is very likely due to the small number of bins with non-zero tweet counts.

**Fig 7:**
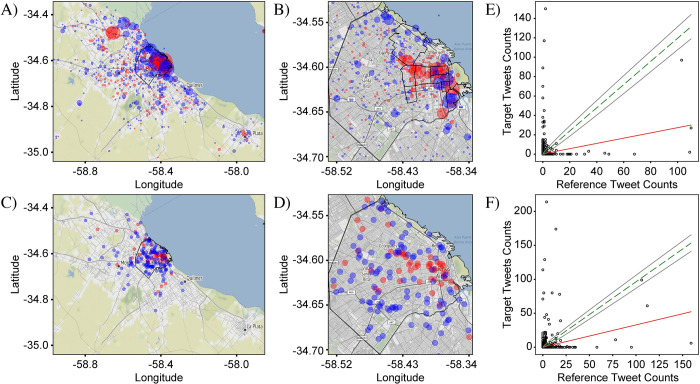
Analysis of “tango” vs. “fútbol”. (A) Differential distribution of words “tango” and “fútbol” in CABA+. Blue (red) circles show where “fútbol” (“tango”) is more prominently tweeted. The use of “tango” is most strongly concentrated in the city, marked by the black outline, and is weak but fairly uniform in the surrounding provinces. For “fútbol”, on the other hand, the contrast between the city and the outskirts is not as severe, and the distribution in the outskirts shows a distinct concentration near the major roadways. (B) Same as (A), but recalculated for CABA. Over this extent, it is clear that the use of “tango” is mostly confined to a region within the upper-right quadrant of the graph which corresponds to the downtown area of Buenos Aires. The outlines of the neighborhoods of La Boca, San Telmo, Palermo, Monserrat, Balvanera, San Nicolás, Almagro and Boedo, known for having many important Tango clubs, are marked in black in the graph, and coincide well with the distribution of red circles. (C) A plot over the extent of CABA+ of soccer-related locations (i.e., soccer fields and stadiums), marked by blue circles, and tango-related locations (i.e., tango schools and tango dance and performance venues), marked by red circles. The locations and their GPS coordinates were identified using Google maps. (D) Same as (C), but zoomed in to the extent of CABA. The agreement between the distributions of “tango” and “fútbol” tweets and the distributions of tango- and soccer-related locations is very good, indicating a strong connection between the two for both extents. (E) and (F) Frequency comparison plots for the data in (A) and (B), respectively. Both show a high degree of anticorrelation indicating a strong driver of variation. CABA+ Statistics: N^T^ = 2,288, N^R^ = 2,327, S_PCC_ = 0.76, KSS = 0.20, GMI = 0.19. CABA Statistics: N^T^ = 1,716, N^R^ = 1,435, S_PCC_ = 0.78, KSS = 0.24, GMI = 0.19. Base map and data from OpenStreetMap and OpenStreetMap Foundation under the Open Database License (Fig 7A–7D).

For the third case (Fig 7), we compare the distributions of tweets mentioning activities *Tango* and *fútbol* (‘soccer’). The differential distributions (Fig 7A & 7B) show a distinctive pattern in which the locations with a greater representation of *Tango*, (in red), occupy a well-defined region in the downtown area. Although the pattern is not as stark as in the case of “La Boca” vs. “Palermo” or the case of the two stadiums, the pattern is nevertheless very clear, especially in comparison to the null cases. A difference between this case and the previous ones is that the words *Tango* and *fútbol* are associated with multiple locations across the city. Tango, for example, is likely to be a topic of discussion in instruction studios as well as in dance venues, whereas soccer will more likely be discussed in soccer stadiums and soccer fields. The distribution of tweets should therefore correspond to the distribution of locations associated with each activity. To check this hypothesis, we used Google Maps to find the locations of the relevant venues and then created maps plotting their distributions, with tango schools and dance venues indicated by red circles and soccer clubs, fields and stadiums indicated by blue circles (Fig 7C & 7D). The pattern of red and blue in the tweet distribution maps match very well with the pattern of tango and soccer venues found on Google Maps. This pattern is also relatively easily explained. The biggest concentration of tango tweets and venues is in the downtown neighborhoods (see Fig 7B), known for many important Tango places. Given that tango is more of a night-time activity, its concentration downtown also makes sense. Soccer a theme of national interest at all levels of society, is represented by tweets in a pattern that is more spread out around the city (Fig 7B). This pattern is even more noticeable on the larger scale of CABA+ (Fig 7A). The strongest concentrations of tweets coincide with specific soccer clubs such as Defensores de Belgrano, Sports Lugano and Boca juniors. The bin-by-bin analysis of the tweet counts (Fig 7E & 7F), shows that the numbers of *Tango* and *fútbol* tweets are mostly anti-correlated, indicating that the two topics occur in distinct regions. The anti-correlation is consistent with a strong signal strength of *S*_*PCC*_ = 0.78, and together with the matching of the spatial patterns of the tweets and the associated venues, indicates that geosocial context plays a strong role in defining the distributions of the two topics.

For the final case, we look at an aspect of language use not related to lexical meaning, but rather to language style: i.e., formality. As style does not generally encode a concept or meaning, it has no direct connection to specific buildings or neighborhoods as in the previous cases. The connection is to a more abstract type of space division, namely public vs. private space. This space division has already been shown to play an important role in how we communicate [1]. It has also been shown that formal language, as represented by, for example, complete and correct sentences, polite forms, technical terms, etc. is used in different social circumstances than informal language, which is more likely to contain incomplete sentences, shortcuts, swear words, and common language terms [1,37]. The former type is more often used in public context situations, such as when addressing an official, where the familiarity relation is missing between the speaker and the addressee [37]. The latter type, on the other hand, is more often used in private and intimate context situations, such as when talking to friends and family [37]. We therefore expect a correlation between formal/informal language and public/private space.

**Fig 8:**
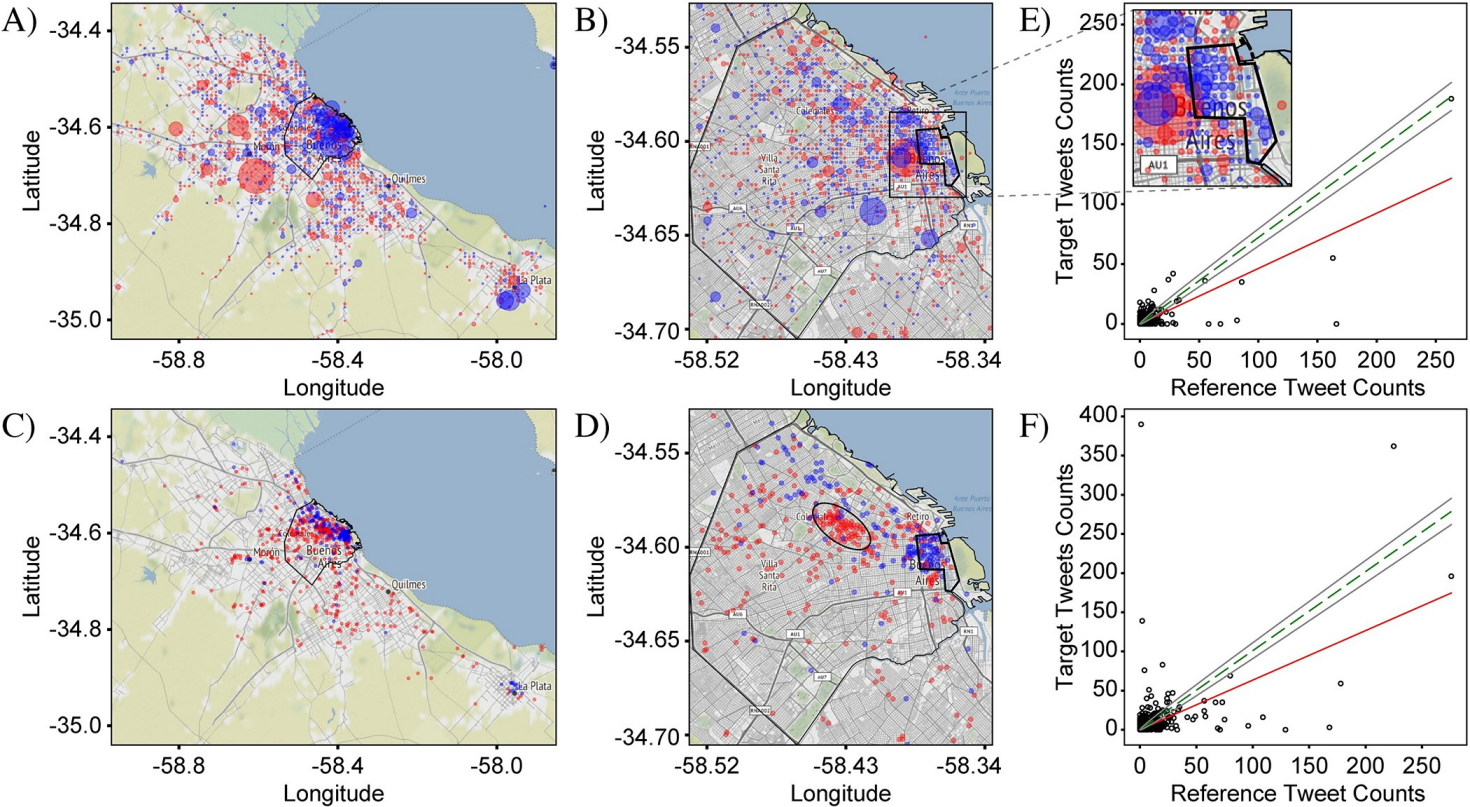
Analysis of informal vs formal tweets. (A) Differential distribution of informal and formal tweets in CABA+. Informal tweets were identified by the presence of one or more of a list of colloquial and vulgar words, while formal tweets were identified as those containing a more formal analogue of the same words. Blue (red) circles show where formal (informal) tweets are more prominent. The distribution shows a higher concentration of formal tweets in the city than in the surrounding areas, while informal tweets are more prominent outside the city. (B) The same as (A), except recalculated for the CABA extent. The distinction between formal and informal is a bit less clear than for the CABA+ extent, but nevertheless, there is a greater prominence of formal tweets in the North-East of the city near the coast, and especially in the Central Business District of Buenos Aires (outlined in black; see also inset of (E)). (C) Plot over the extent of CABA+ of locations with a formal character, including banks, government buildings, ministries, consulates, embassies, courts and law offices, marked by blue circles and bars, representing informal locations, marked by red circles. The distribution of formal and informal locations matches well with the distribution of formal and informal tweets in (A), with a strong concentration of formality in the North-East of the city, and more distributed informality in the rest of the city and the surrounding areas. (D) Same as (C), but zoomed in to the extent of CABA. The distribution of formal locations matches well with that of formal tweets in (B), especially in the Central Business District and along the coast The wide spread of informal locations matches that of informal tweets in (B), but the concentration in the center, marked by the black oval, which corresponds to the bar district of Buenos Aires does not. The discrepancy indicates that bars are not an exclusive context for informality, which is not surprising. (E) and (F) Frequency comparison plots for the data in (A) and (B), respectively. These blots show a moderate degree of anticorrelation, indicating the presence of a driver of variation. CABA+ Statistics: N^T^ = 5,301, N^R^ = 5,251, S_PCC_ = 0.4526, KSS = 0.1128, GMI = 0.0679. CABA Statistics: N^T^ = 2,348, N^R^ = 3,254, S_PCC_ = 0.4519, KSS = 0.0674, GMI = -0.1046. Base map and data from OpenStreetMap and OpenStreetMap Foundation under the Open Database License (Fig 8A–8D).

Within a city, public space includes those associated with commerce, finance, administration and government, for example, which tend to be concentrated near the city center. Services in the form of tweets addressed to the general public, such as weather forecasts, official news from publishing services, commercial services, etc., are therefore more likely to come from the city center. Private space, on the other hand, is more likely to include areas associated with leisure activities as well as with residential zones, which tend to be distributed farther away from the city center. To test this hypothesis, we plotted the differential distribution of informal vs formal tweets over the extents of CABA+ and CABA (Fig 8). For this analysis, we define informal tweets as those containing vulgar or swear words, e.g. *mierda* ‘shit’ and corresponding formal words or medical words that have the same meaning or refer to the same object as informal or swear words, such as *excremento* ‘excrement’ (see S3 File). On the CABA+ extent (Fig 8A), the graph shows a relatively clear partitioning of the space into downtown and peripheral areas. The regions favoring formal language (shown in blue) are strongly concentrated in a relatively small region within the downtown area of the city, while the surrounding suburban and rural zones are dominated by informal language (red).

Recalculating the distribution on the smaller area of CABA, we see less of a clear division between formal and informal, indicating that within the boundaries of the city there is a greater mixture of public and private space. Searching for government offices using the street-view feature of Google Maps [38], one finds, in fact, that it is not uncommon for these government offices to be collocated with residential space in tall apartment buildings. Nevertheless, a concentration of tweets tagged as formal can be found in the upper-right quadrant of Fig 8B corresponding to the area known as the “Central Business District” (outlined in black and within inset of Fig 8E). This area is known to be the home of many historical, governmental and political buildings, comprises the banking head-quarters of the country and coincides with the historic center of the Plaza de Mayo as well as many other buildings of political and cultural importance such as the presidential palace *La Casa Rosada*.

For comparison, in Fig 8C & 8D, we created maps of public and private space by plotting the locations (obtained from Google Maps) of buildings of the appropriate character. To define public locations (marked with blue circles), we used financial buildings, such as banks and currency exchanges; governmental buildings, such as embassies, ministries and administrative centers; and judicial buildings, such as courts and attorney offices. Private locations (marked with red circles) were defined using the locations of bars. On the CABA+ extent (Fig 8C), the pattern of public and private space matches reasonably well with the pattern of formal and informal tweets (Fig 8A), i.e., public/formal locations are predominantly located within a small subset of the city near the water’s edge and private/informal locations widely distributed over the whole extent. On the CABA extent (Fig 8B & 8D), there is still a noticeable concentration of blue near the water’s edge and red distributed more widely. However, the map of public and private space (Fig 8D) shows a greater degree of separation than the map of formal and informal tweets (Fig 8B) and the strong concentration of bars (marked by a black oval in Fig 8D) does not have a corresponding feature in Fig 8B, indicating that vulgarity may not be a perfect proxy for informality. Nevertheless, the separation observed, over both extents, in the spatial distributions of the two tweet types agrees well with the model that public spaces tend to favor more formal language and private space is relatively more likely to have informal language. The corresponding frequency-comparison plots (Fig 8E & 8F) shows that counts of formal and informal tweets tend to anti-correlate, confirming the presence of a measurable signal.

The four cases presented above give strong evidence that the influence of geosocial context on tweeting behavior can be measured. The influence is very strong when the “tweeting behavior” corresponds to mentioning the locations people are tweeting from, and can also be clearly distinguished in the more abstract case where the tweeting behavior corresponds to a style of language use.

### Signal variations from language use connected to social groups

In this section, we investigate a complementary aspect of language variation driven by social groups. We define social groups, here, very generally as any group of people with a distinguishing characteristic. We examine three different cases with contrasting pairs of social groups and look for the presence of measurable spatial patterns in their tweeting locations.

**Fig 9:**
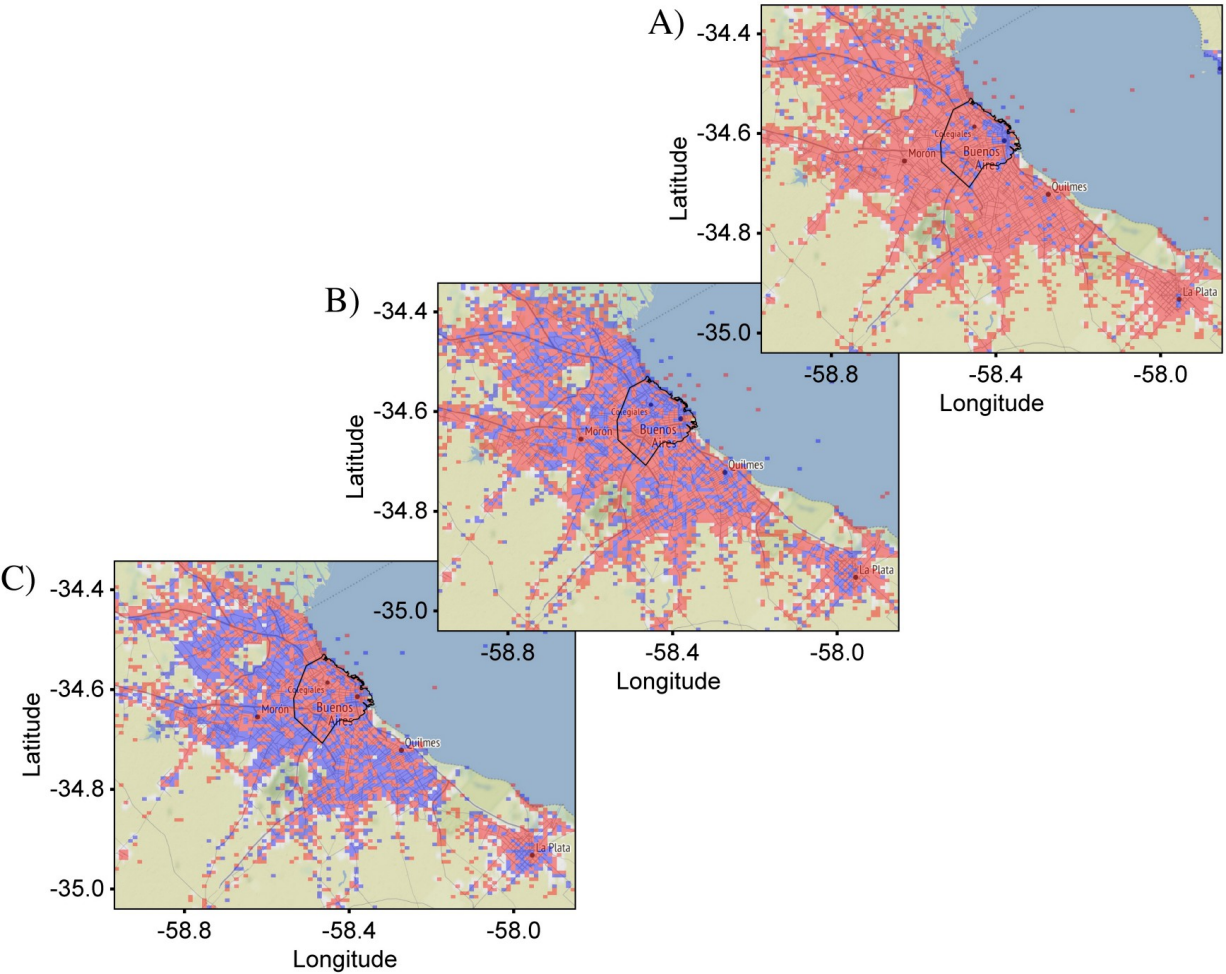
Comparison of differential distributions for three user cases with aspects of “foreignness” plotted over CABA+. (A) Foreign vs. local users. Blue represents the locations from which foreign users (i.e., from foreign countries) tweet more prominently while red represents the locations from which local users (i.e., from CABA+) tweet more prominently. (B) English vs. Spanish users. Blue (red) represents the locations from which English (Spanish) users tweet more prominently. (C) PanSp vs. ArgSp users. Blue (red) represents the locations from which Peninsular Spanish dialect users (Argentinian Spanish dialect users) tweet more prominently. The three cases show highly distinct patterns indicating that the users in each case and the corresponding geosocial drivers are most likely also distinct. Base map and data from OpenStreetMap and OpenStreetMap Foundation under the Open Database License.

The three cases of social groups we have chosen have a common theme in that for each pairing, one of the social groups has an aspect of being local and the other an aspect of being foreign. Case 1 studies the effect of user origin. The local social group is the set of users who indicate in their Twitter account a user location belonging to the greater metropolitan area of Buenos Aires, while the foreign user group is the set of users indicating their user location as a foreign country. For both groups, the language of the tweets is restricted to Spanish. Case 2 studies the effect of user language. The “local” users are those who tweet in Spanish, while the “foreign” users are those who tweet in English. The selection of users is done by collecting the set of unique users (as identified by their user ID) who have tweeted at least once in each language, and the full set of tweets belonging to each user is then used for the analysis. A consequence of this approach is that users who tweet in both languages are counted twice. Case 3 studies the effect of user dialect. “Local” users are those who tweet with the primary, or standard local dialect, Argentinian Spanish (ArgSp), while “foreign” users are those who tweet using the dialect spoken on the Spanish peninsula and in a number of other Latin-American countries (PanSp), referred to in the traditional terminology as the “pan-hispanic” variety [22,39,40] (see analysis of Case 3 for a more complete definition of ArgSp and PanSp). The selection of users and tweets is done similarly as in Case 2. To provide an overview of the three cases, Fig 9 shows the differential distributions of tweets plotted over the extent of CABA+, with the administrative borders of CABA shown in outline.

Cases 1–3 show a distinct progression. In Case 1 (foreign vs. local users, Fig 9A), the area in blue, designating the region where foreign users are most represented, is highly concentrated in the downtown area. Outside of this high-concentration region, there is very little representation of foreigners in the whole of the CABA+ extent. In Case 2 (English vs. Spanish users, Fig 9B), as English is the lingua franca for tourist and business communication, our initial hypothesis was that English users would have a similar distribution as foreign users, concentrating downtown vs the periphery of CABA. The results only partially match our expectations, with some concentration in touristic places but a much greater distribution throughout the city, suggesting that English users are more integrated into the city than foreign users in general. In addition, there is a noticeable preference for English users in the North, which will be discussed further below. By contrast, Case 3 (PanSp vs. ArgSp dialects, Fig 9C) shows a completely different pattern. The concentration is reversed, in that there is a greater amount of blue, representing a relative prominence of the PanSp dialect users, in the outer provinces of CABA+ compared to the city.

Comparison of the patterns observed for the three cases of social groups give us several important pieces of information. First, choosing different sets of users results in different differential distribution plots, indicating that it is, indeed, possible to measure the spatial patterns of language use associated with social groups on the urban scale. Second, we learn that foreigners, English speakers and PanSp speakers are not the same groups of people, which suggests that locals communicate not only in their standard dialect of ArgSp, but also in PanSp and in English. Each of these three cases is explored in more detail, individually, below.

**Fig 10:**
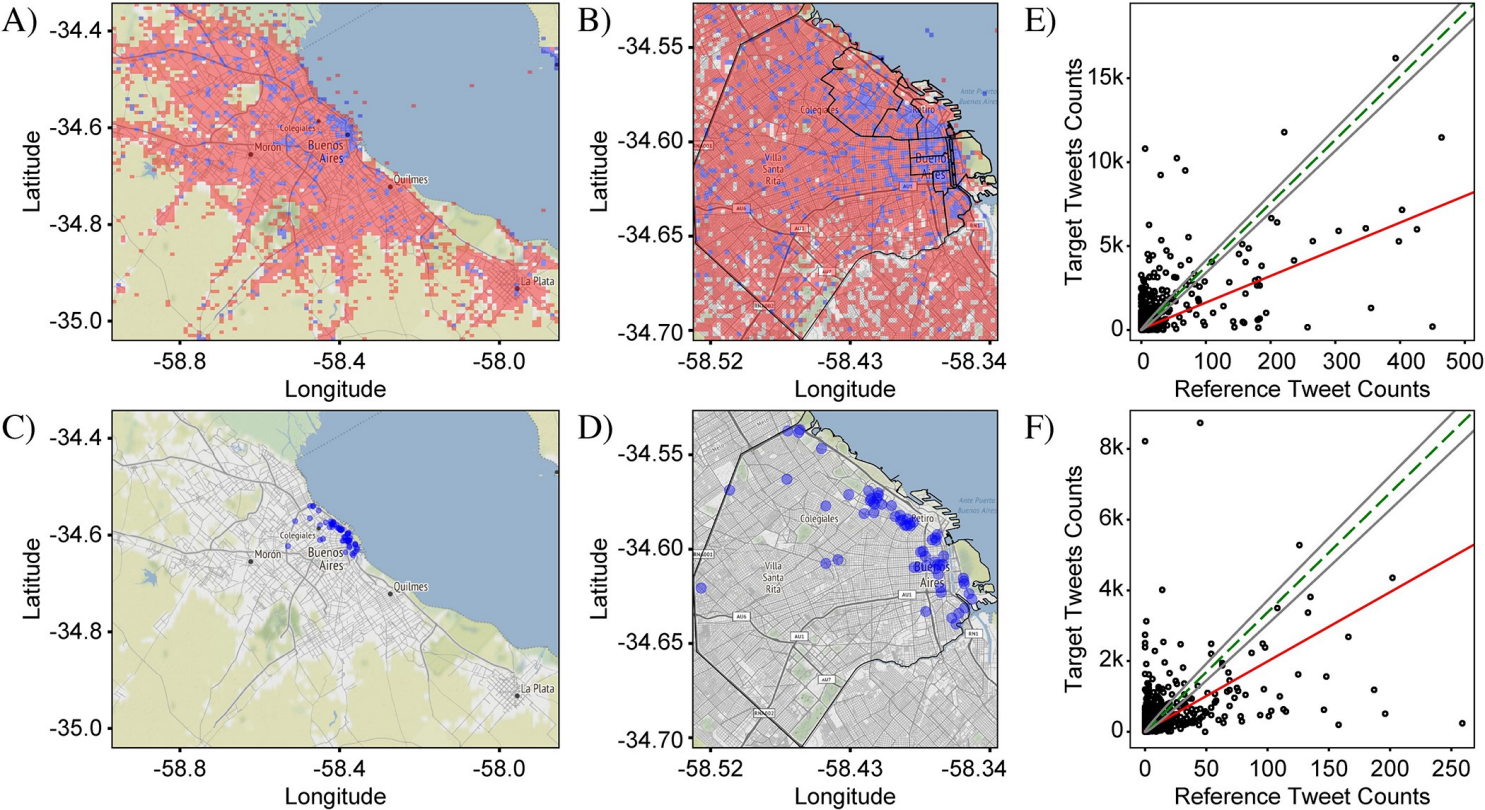
Analysis of local vs. foreign users. (A) & (B) Differential distributions of local vs. foreign users over CABA+ and CABA extents, respectively. Blue (red) indicates where foreign (local) users tweet more prominently. The graph in (A) shows that foreign users are much more strongly represented within the city than in the surrounding area, while the graph in (B) shows that they are mostly localized to the eastern and north-eastern part of the city along the coast in the neighborhoods of Palermo, Recolleta, Retiro, San Nicolás, Monserrat, Puerto Madero, San Telmo and La Boca, outlined in black. (C) & (D) Maps of the locations of monuments, museums and other attractions extracted from Google Maps, plotted over the extents of CABA+ and CABA, respectively. The pattern of these attractions matches very well with the pattern of tweeting by foreign users both on the extents of CABA+ and CABA, indicating tourism as a strong candidate as a driver of the pattern. The highest concentration of blue bins is in the area of the Central Business District, identified in Fig 8B, which is known as the cultural and business center of Buenos Aires [42], indicating business as another potential driver. (E) & (F) Frequency comparison plots for CABA+ and CABA, respectively. The plots show a wide dispersion of points around the exact-correlation line, indicating the presence of moderately strong signal. The dispersion appears slightly greater in the CABA+ than in the CABA data, which agrees with CABA+ having a stronger signal, as measured by both the Pearson correlation and Kolmogorov-Smirnov statistic-based metrics. CABA+ Statistics: N^T^ = 764,661, N^R^ = 20,237, S_PCC_ = 0.22, KSS = 0.17, GMI = 0.11 CABA Statistics: N^T^ = 442,638, N^R^ = 13,106, S_PCC_ = 0.07 KSS = 0.11, GMI = 0.04. Base map and data from OpenStreetMap and OpenStreetMap Foundation under the Open Database License (Fig 10A–10D).

For Case 1 (foreign vs. local users, Fig 10), the pattern of blue concentration observed in Fig 9A (and reproduced in Fig 10A) is now resolvable to the neighborhoods of Palermo, Recolleta, Retiro, San Nicolás, Monserrat, Puerto Madero, San Telmo and La Boca, whose borders have been outlined in Fig 10B. These are among the most visited neighborhoods in CABA [41] and are popular tourist destinations. This region also has a large overlap with the *Microcentro* (identified in Fig 8B) which includes the financial and governmental centers of the city, as mentioned in the previous section. This pattern suggests that tourism, business and foreign affairs are important activities that characterize foreigners, which is not unexpected. To confirm the role of tourism in determining the patterns in case 1, we plot the distribution of museums, monuments and other tourist attractions on both extents (Fig 10C & 10D) using locations obtained from Google Maps. The similarity between the patterns of blue in the differential distributions and the spatial arrangement of attractions is very clear, strengthening the connection between foreigners and finances/business/tourism. The corresponding frequency comparison plots (Fig 10E & 10F) for CABA+ and CABA, respectively show that the counts of local and foreign tweets are not strongly correlated, indicating the presence of a robust variation signal. As a side note, the clear localization of tweets from foreign users and the correspondence with tourist locations implies that the “user location” metadata is, at least to some degree, reliable.

**Fig 11:**
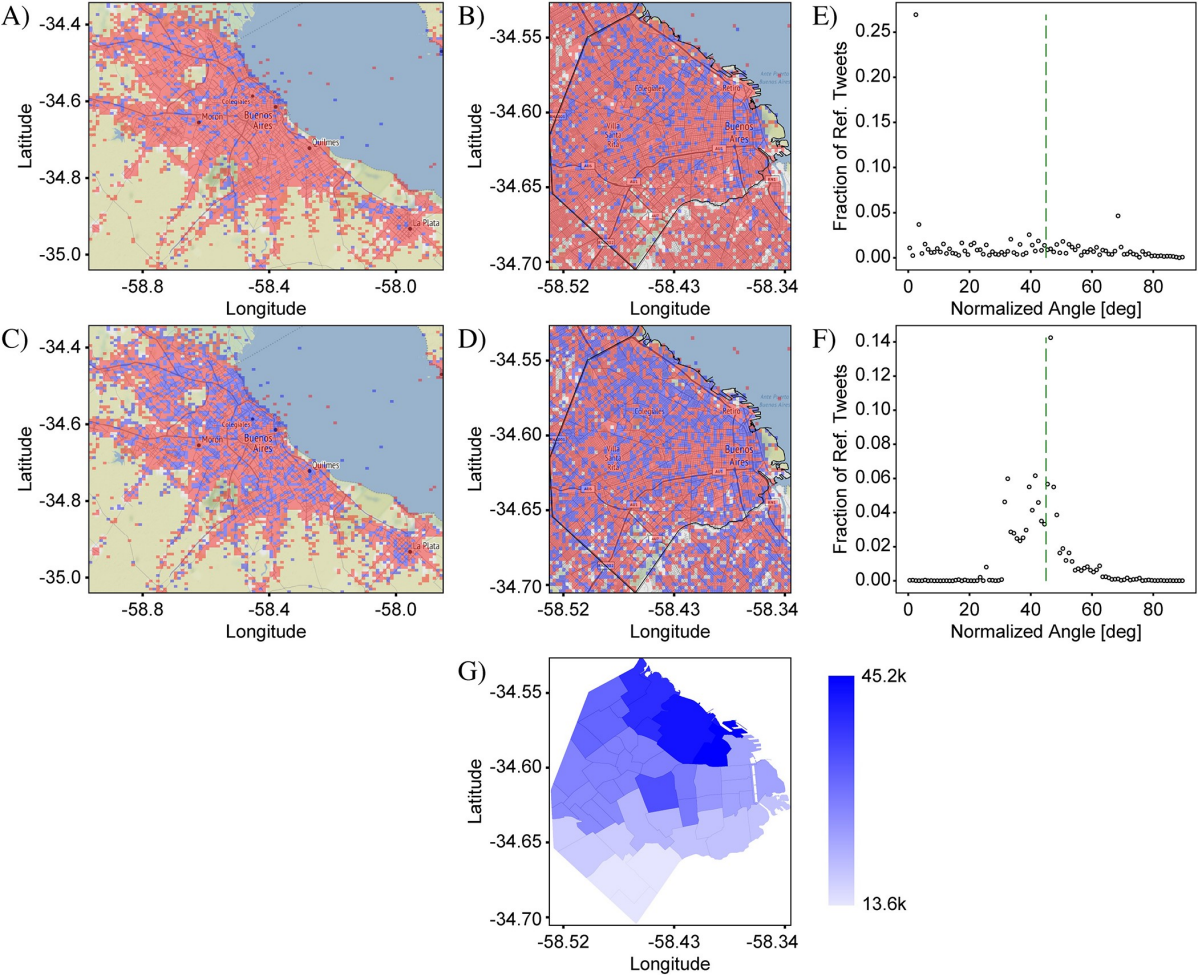
Analysis of Spanish vs. English. (A) & (B) Differential distributions of Spanish vs. English tweets over CABA+ and CABA extents, respectively. Blue (red) indicates where English (Spanish) tweets are more prominent. The graph in (A) shows that there is a noticeable preference for English in the northern part of CABA+ (upper half of the graph) as well as closer to the city. The north-south pattern can also be seen in (B), where the number of blue bins increases from bottom to top. (C) & (D) Differential distributions of all tweets generated by the users from (A) and (B), respectively. Blue (red) indicates where users associated with the English (Spanish) tweets tweet more prominently. A north-south asymmetry is still observable, but is weaker than in (A) and (B). Overall, the relative distribution of the English and Spanish users shows clear differences to that of the English and Spanish tweets themselves, indicating that users tweet in both languages. Graphs in (A) and (B) therefore describe the geographic pattern where Spanish and English languages are used (i.e., the *geosocial context* associated with these languages), whereas the graphs in (C) and (D) describe geographic patterns associated with users of the language (i.e., the *social groups*). The conclusion above that the users are bilingual (i.e., tweet in both languages) also suggests geographic patterns defined by the users will be weaker than those defined by the tweets. (E) & (F) Normalized angle histograms associated with (B) and (D). (E) shows a large spread of angles, indicating anticorrelation between English and Spanish tweet counts, or a strong driver of variation. By contrast, (F) shows a very narrow spread of angles, indicating a strong correlation between English and Spanish users, or a lack of driver of variation. These results agree with the predictions from the comparison of the differential distributions. (G) Average monthly income per capita by county. This graph shows a gradient in wealth from south to north which matches well with the pattern of blue in (B). This correspondence hints at a correlation between the use of English and wealth. CABA+ Statistics (Sp. vs. Eng. Users): N^T^ = 1,228,139, N^R^ = 744,539, S_PCC_ = 0.0807, KSS = 0.0347, GMI = 0.0045. CABA Statistics (Sp. vs. Eng. Users): N^T^ = 718,280, N^R^ = 438,086, S_PCC_ = 0.0153, KSS = 0.0332, GMI = 0.0164. CABA+ Statistics (Sp. vs. Eng. Tweets): N^T^ = 1,151,785, N^R^ = 118,231, S_PCC_ = 0.7236, KSS = 0.1409, GMI = -0.0055. CABA Statistics (Sp. vs. Eng. Tweets): N^T^ = 667,365, N^R^ = 62,746, S_PCC_ = 0.7128, KSS = 0.1385, GMI = -0.0045. Base map and data from OpenStreetMap and OpenStreetMap Foundation under the Open Database License (Fig 11A–11D). Census data and shape files for Fig 11G obtained from the official website of Buenos Aires [44,45].

For Case 2, (Spanish vs English users), we perform a different kind of analysis only made possible by the ability to connect multiple tweets to a particular user, enabled by the presence of “User ID” metadata (Fig 11). This analysis compares how users tweet particularly in Spanish or English compared to how they tweet in general. We identify Spanish users by collecting all the unique user IDs from our Spanish corpus (i.e., all geolocated Spanish tweets from a given extent), and similarly, we identify English users by the unique user IDs from the English corpus. In Fig 11A & 11B, we plot the differential distributions (across CABA+ and CABA, respectively), for Spanish tweets tweeted by Spanish users compared to English tweets tweeted by English users. Due to the definitions above, this is equivalent to the differential distribution of all Spanish tweets vs all English tweets. In Fig 11C & 11D, however, we plot the differential distribution of all tweets (from both Spanish and English corpora) tweeted by the Spanish users vs all tweets from both corpora tweeted by the English users. Several differences can be seen between these two types of differential distribution. In the first variant, “Spanish vs English tweets”, there is a preference for English in the northern half of the graphs which is quite clear. By contrast, for the second variant, “Spanish vs English users”, the prominence for English is still noticeable, but not as clear, with the blue squares more evenly distributed over the extents. In Fig 11E & 11F, we plot the normalized angle histograms for the two versions, which show that in addition to a difference in the clarity of the spatial pattern, there is also a much stronger signal in the Spanish vs English tweets variant (1-r = 0.715, where r is the Pearson correlation coefficient) than in the Spanish vs English-users variant (1-r = 0.015). The angle histograms were presented here instead of the frequency comparison plots as they show this effect more clearly.

If each user tweeted in only one language, there would be no difference between the two variants. Therefore, logically, the presence of noticeable differences in the distributions and statistics for the two cases necessitates that a large fraction of the users is tweeting in both languages. We can confirm this conclusion directly from the data. The total number of Spanish and English users is 140,019 and 27,753, respectively. The number of users who have tweeted at least once in both languages (which we henceforth refer to as “bilingual users”), is 20,463. In other words, 5% of the Spanish users are bilingual, while 75% of the English users are bilingual. These statistics give us several important pieces of information. First, to a rough approximation, the English users are a subset of the Spanish users. Second, English is only used by a small fraction of the Spanish users, indicating this fraction as being specialized in some regard. Finally, the English users, being capable of tweeting in both languages, have the ability to choose when and where to use each language. For example, in Fig 11B, there is a stronger concentration of blue in the *Central Business District* identified in Fig 8B, suggesting that English is connected to business, which is well known.

A study performed by Würth [43] connects the use of English words in Spanish in Buenos Aires to wealth, which is found to be more concentrated in the North. Using census data obtained from the official website of Buenos Aires [44,45], we confirm that within CABA the average monthly per capita income indeed displays a South-North gradient (Fig 11G), reinforcing a possible connection between English use and wealth. According to business and economy reports, this pattern is also present in the urban periphery of Buenos Aires or CABA+ [46]. Würth [43] concludes that this connection is due to an increased likelihood of English-word use by wealthier people. However, our study suggests that wealth has a much stronger effect on the use of English and not so much on the distribution of English users. The fact that the *Spanish vs English tweets* variant shows both a higher degree of clarity in the spatial pattern as well as a higher signal strength than the *Spanish vs English users* variant indicates that the geosocial context plays a stronger role than the social group in determining the tweet behavior. Following this logic, as English is a foreign language and also the lingua franca for tourist and business communication, one might expect that (similarly to Case 2 comparing local vs foreign users) its use would be concentrated in areas associated with tourism and commerce (identified in Fig 10). We find, however, that while English is well represented in those areas, the pattern of English tweets is much more widely distributed across the city than the pattern of foreign users.

**Fig 12:**
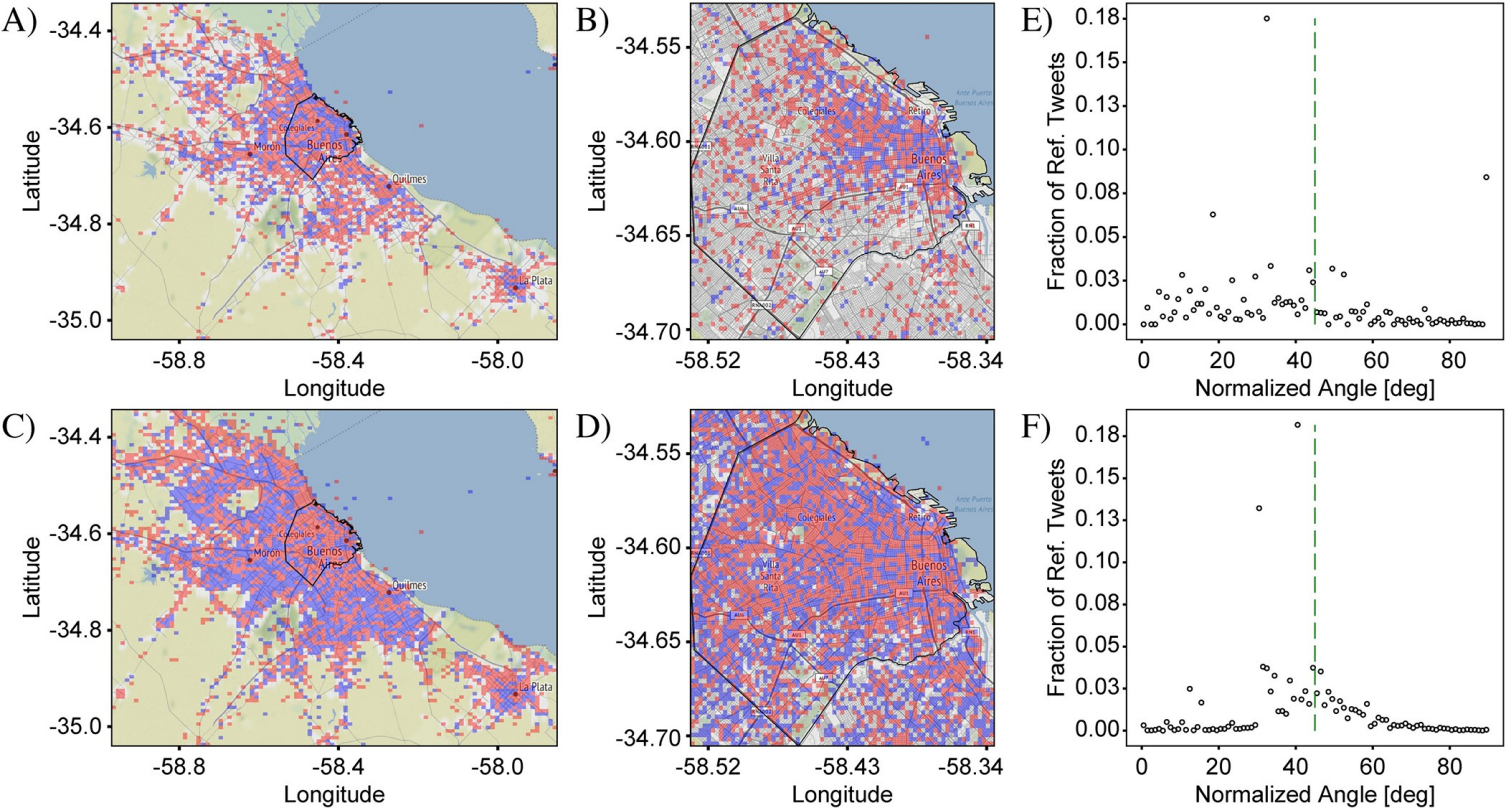
Analysis of PanSp vs. ArgSp users. (A) & (B) Differential distributions of PanSp vs. ArgSp tweets over CABA+ and CABA extents, respectively. Blue (red) indicates where PanSp (ArgSp) tweets are more prominent. The graph in (A) does not show a strong distinction between the city and the surrounding areas. The most convincing feature is arguably the strip of blue parallel and close to the shore. This cluster of blue bins is similar in shape to the pattern of tourist attractions presented in Fig 10(C), suggesting that tourism may play a role in determining the use of PanSp. The graph in (B), however, does not show a distinctive pattern, showing that the choice of dialect is not strongly determined by location within the city. It should be noted, however, that the overall count of ArgSp tweets outweighs that of PanSp tweets, marking the former as the more standard. (C) & (D) Differential distributions of tweets by users associated with PanSp tweets vs tweets by users associated with ArgSp tweets from (A) and (B), respectively. As in the English vs Spanish case, clear differences exist between the tweet-defined relative distribution and the user-defined one, highlighting the distinction between the geosocial contexts and the social groups associated with the two dialects. In particular, the graph in (C) shows a clear preference by PanSp users for the surrounding areas of the city versus the city itself. The graph in (D), however, shows that within the city there is no strong spatial pattern in the users of PanSp. It should be noted that tweets containing PanSp or ArgSp tokens are a small subset of the total tweet corpus. As a result, the inclusion of all tweets by the users increases the counts considerably. (I) & (F) Normalized angle histograms of the data in (B) and (D). As in the English vs Spanish case, the tweet-defined counts show a much weaker correlation than the user-defined counts indicating that despite the lack of a strong spatial pattern, a geosocial context influencing the choice of dialect does seem to exist. CABA+ Statistics (ArgSp vs. PanSp Users): N^T^ = 594,312, N^R^ = 345,841, S_PCC_ = 0.03, KSS = 0.02, GMI = -0.05. CABA Statistics (ArgSp vs. PanSp Users): N^T^ = 332,455, N^R^ = 192,621, S_PCC_ = 0.02, KSS = 0.04, GMI = 0.009. CABA+ Statistics (ArgSp vs. PanSp Tweets): N^T^ = 18,731, N^R^ = 5,607, S_PCC_ = 0.10, KSS = 0.05, GMI = -0.004. CABA Statistics (ArgSp vs. PanSp Tweets): N^T^ = 10,113, N^R^ = 3,324, S_PCC_ = 0.06, KSS = 0.05, GMI = 0.02. Base map and data from OpenStreetMap and OpenStreetMap Foundation under the Open Database License (Fig 12A–12D).

For Case 3 (ArgSp vs PanSp dialects) we perform a similar analysis as for Case 2 (Fig 12). The dialects are defined on the basis of a very well-known linguistic distinction in the pronoun forms of the informal 2^nd^ person singular ‘you’ and present tense indicative verbs that agree with the 2^nd^ person singular (e.g., ArgSp *vos tenés* vs. PanSp *tú tienes* ‘you have’) [22,39,40]. Moreover, we included well-known lexical words that refer to the same concept such as ArgSp *Che* vs. PanSp *Oye* ‘Hey!’ [23]. The complete list can be found in (S3 File).

Fig 12A & 12B show the differential distribution for ArgSp vs PanSp tweets over the extents of CABA+ and CABA, respectively. In Fig 12C & 12D, the differential distributions are recalculated by including all of the tweets written by the users (i.e., “ArgSp vs PanSp users”). Comparison between the *ArgSp vs PanSp tweet* distributions and the *ArgSp vs PanSp user* variants leads to several important points. First, we see that there are distinct differences between the two types of distributions indicating that the locations where users tweet in a particular dialect are not the same as where the users tweet in general. This discrepancy has a number of important implications. First, since the number of tweets containing tokens that unambiguously determine the dialect is only about 1% or less of the total, one might think to improve efficiency of dialect assignment (thereby increasing the dataset) by using the unambiguous tweets to identify users and then taking all of the tweets of ArgSp users to be ArgSp tweets and all of the tweets of PanSp users to be PanSp tweets. The differences between the user and tweet distributions show that this procedure would give false results. We can also conclude, just as with the Spanish vs English case, that a large number of users tweet in both dialects. Directly counting the users, we find, in fact, that out of 12,681 ArgSp users and 4,954 PanSp users, there are 1,828 users tweeting in both dialects (whom we henceforth refer to as “bidialectal users”).

These numbers, together with the differential distributions allow us to make inferences about the role of the two dialects within the society of Buenos Aires. The fact that there are more than 3x as many ArgSp as PanSp tweets in the corpus (i.e., 18,731 vs 5,607) is consistent with the established fact that ArgSp is the standard and official dialect [22,39]. Given this status, and given that Spanish-speaking foreigners typically use PanSp, one might associate PanSp with foreigners and tourism. However, unlike the foreigner tweets from Case 2 which are highly localized, PanSp tweets are widely distributed throughout the city, in particular, *outside* the touristic hotspots. Fig 12A & 12B show, in fact, that the representation of ArgSp and PanSp tweets is nearly uniform over both extents. For ArgSp vs PanSp *users* variant, on the other hand, while the differential distribution over the CABA extent (Fig 12D) shows a similar pattern of uniformity, the *users* distribution over the CABA+ extent (Fig 12C) shows a clear pattern of over-representation of PanSp *users* on the outskirts of the city as well as a slight prevalence in the South compared to the North. These patterns very clearly do not coincide with those of tourists or users of foreign origin (as seen in Case 2) and therefore show that a large fraction of PanSp users must therefore be locals. This conclusion indicates that despite not being the standard dialect, PanSp is nevertheless an integral part of the dialectal landscape of the city. The fact that a large fraction of ArgSp and PanSp users are bidialectal (i.e., 14% and 37%, respectively) reinforces the above conclusion.

This discrepancy between the user and tweet distributions is also important in that it strongly indicates that a geosocial context exists which influences where each dialect is used. Both the S_PCC_ and KSS signal-strength metrics as well as the normalized histograms in Fig 12E & 12F show, in fact, that a stronger signal exists for *ArgSp vs PanSp tweets* than for *ArgSp vs PanSp users*. A careful inspection of Fig 12A reveals that over the CABA+ extent, there is a perceptible preference for PanSp tweets within the city and especially in the region along the coast associated with tourist destinations (see Fig 10 and associated analysis). This pattern hints that while PanSp is a dialect used by locals, it is, at least in certain circumstances, more likely to be used *by* tourists or *in communication with* tourists. A definitive explanation of the patterns in Fig 12 clearly requires a more detailed study connecting the results to the known social structure of the city. One interesting direction, for example, is to look for correlations between dialect use and socioeconomic and educational status, the latter of which have been shown to exhibit patterns closely resembling the one in Fig 12C [47]. This work is beyond the scope of this article. Nevertheless, despite extensive previous analysis of the dialect structure in Buenos Aires [22,39], the wide-scale integration of PanSp into the life of Buenos Aires as well as the presence of a geosocial context influencing its use have not been previously reported and are, in themselves, significant findings.

### Statistical comparison of all cases

As we have seen in the discussions above, comparison of similar cases enables a more robust interpretation of the complex features in the differential distributions by providing a frame of reference for the evaluation. In a similar vein, we here perform several comparisons of the statistical analyses for the complete set of cases and use the fact that they span the full range of signal strength and pattern clarity to bring perspective into the interpretation of the various graphs and metrics we have employed.

**Fig 13:**
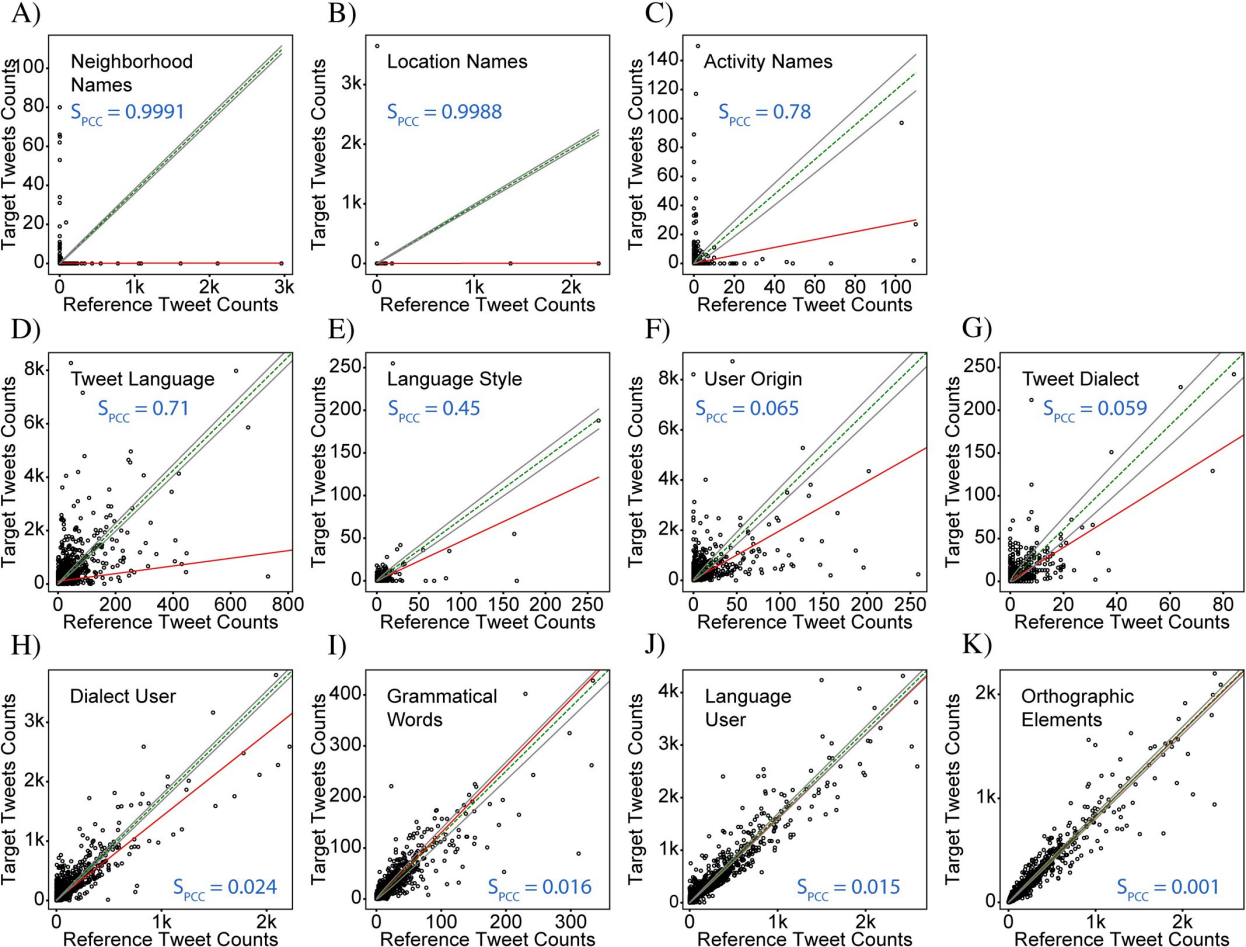
Frequency-comparison plots for the eleven categories of linguistic features. Plots are arranged in order of descending signal strength (S_PCC_) from left to right and from up to down. The green-dotted line represents the *exact-correlation* line, the two grey lines on either side of it indicate the expected range of random statistical variation, and the red line is the linear best fit. For this analysis, the ranges of the x and y axes have been adjusted in order to zoom into the dense part of the plot, allowing for better interpretation of the distribution of points. The sequence of plots shows a pattern of transition from anti-correlation at high signal strength to correlation at low signal strength. Three categories of plot are evident. In the first row, the points have a binary distribution, indicating that tweeters in each location wrote exclusively target or exclusively reference tweets, but not both. This type of distribution shows that the geosocial context had a very strong impact on the tweeting behavior. In the second row, the points demonstrate a large dispersion around the *exact-correlation* line, indicating that at each location there was a mixture of tweet variants, with the degree of mixing varying somewhat continuously from 0–100% of either variant. This type of distribution shows that geosocial context had a moderately strong influence on tweeting behavior. In the third row, the points are mostly clustered around the *exact-correlation* line, indicating small deviations from equal representation of the two variants. This distribution shows geosocial context had a small impact on tweeting behavior. This analysis shows that the shape of the frequency-comparison plot provides important information that elucidates the nature of the geosocial context and the resultant geosocial driver.

Fig 13 shows the frequency-comparison plots (using the CABA extent) for the eleven categories arranged in order of ascending *Pearson correlation coefficient* (i.e., decreasing signal). A number of important observations can be drawn from the comparison. The most obvious is that the plots show a clear pattern of transition from full anti-correlation for Pearson coefficients near zero (very strong signal) to full correlation for Pearson coefficients near one (very weak signal). More interestingly, the way the frequency-comparison plots change during this transition is not just quantitative, but also qualitative, varying from inverse linear to linear in character. In the very-strong-signal cases (Fig 13A–13C), the tweet-distribution is binary, with each location (bin) having exclusively target or reference tweets, but not both. The geosocial context for these cases thus clearly has a very strong effect on tweeting behavior. Meanwhile, for the less-strong-signal cases (Fig 13D–13G) the bins have a mixture of the two tweet variants, with the degree of preference for one variant or the other (as indicated by deviation of the points from the *exact-correlation* line) ranging continuously from zero (equal preference) to 100% (exclusive preference). In these cases, it is clear not only that the geosocial context effects the tweeting behavior only up to a certain degree, but also that the strength of the effect varies from location to location. Finally, for the low-signal cases (Fig 13H–13K), most locations show very little preference for either tweet variant, indicating little or no effect of geosocial context on tweeting behavior. The consistency between the calculated signal strength and the tweeting behavior validates the use of the Pearson coefficient as a signal metric. This analysis also shows that the shape of the frequency-comparison plot tells us something about the nature of the geosocial driver (i.e., whether the impact on the tweeting behavior can be characterized in a continuous or a binary way, and if continuous, to what extent).

**Fig 14:**
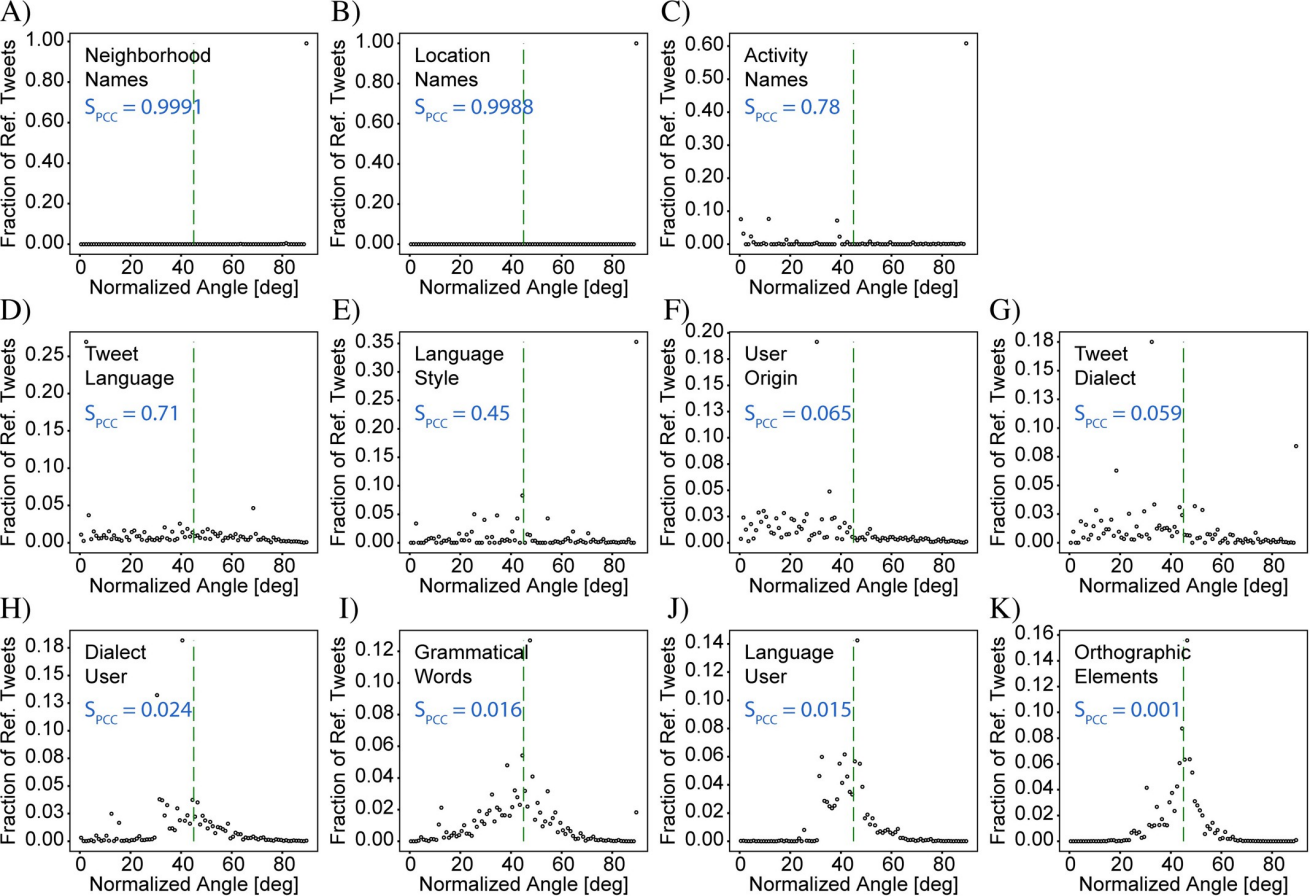
Normalized angle histogram plots for the eleven categories of sociolinguistic features. Plots are arranged in order of signal strength (S_PCC_) from left to right and from up to down. The three rows of graphs correspond to three categories of signal strength, with very-strong-signal cases having perfectly flat histograms, intermediate-strength-signal cases having a very wide distribution, and low-strength-signal cases exhibiting a normal distribution. The correspondence between the shape of the histograms and the signal strength enables an intuitive interpretation of the various cases.

Fig 14 shows the corresponding set of normalized angle histograms arranged in the same order. Once again there is a clear transition from completely flat distributions of angles to strongly-peaked ones as the Pearson coefficients go from zero to one. The three categories of data observed in the frequency-comparison plots are also apparent here. The first row shows angular histograms that are perfectly flat, the second row shows rather even histograms with amplitude over a wide range of angles, while the third row shows histograms with a clear peak around the exact-correlation point (45 degrees). The good correspondence between the shape of the angle histograms, the features of the frequency-comparison plots and the values of the S_PCC_ signal-strength metric shows that the angle histograms provide a valuable, intuitive tool for quick interpretation of the signal strength.

**Fig 15:**
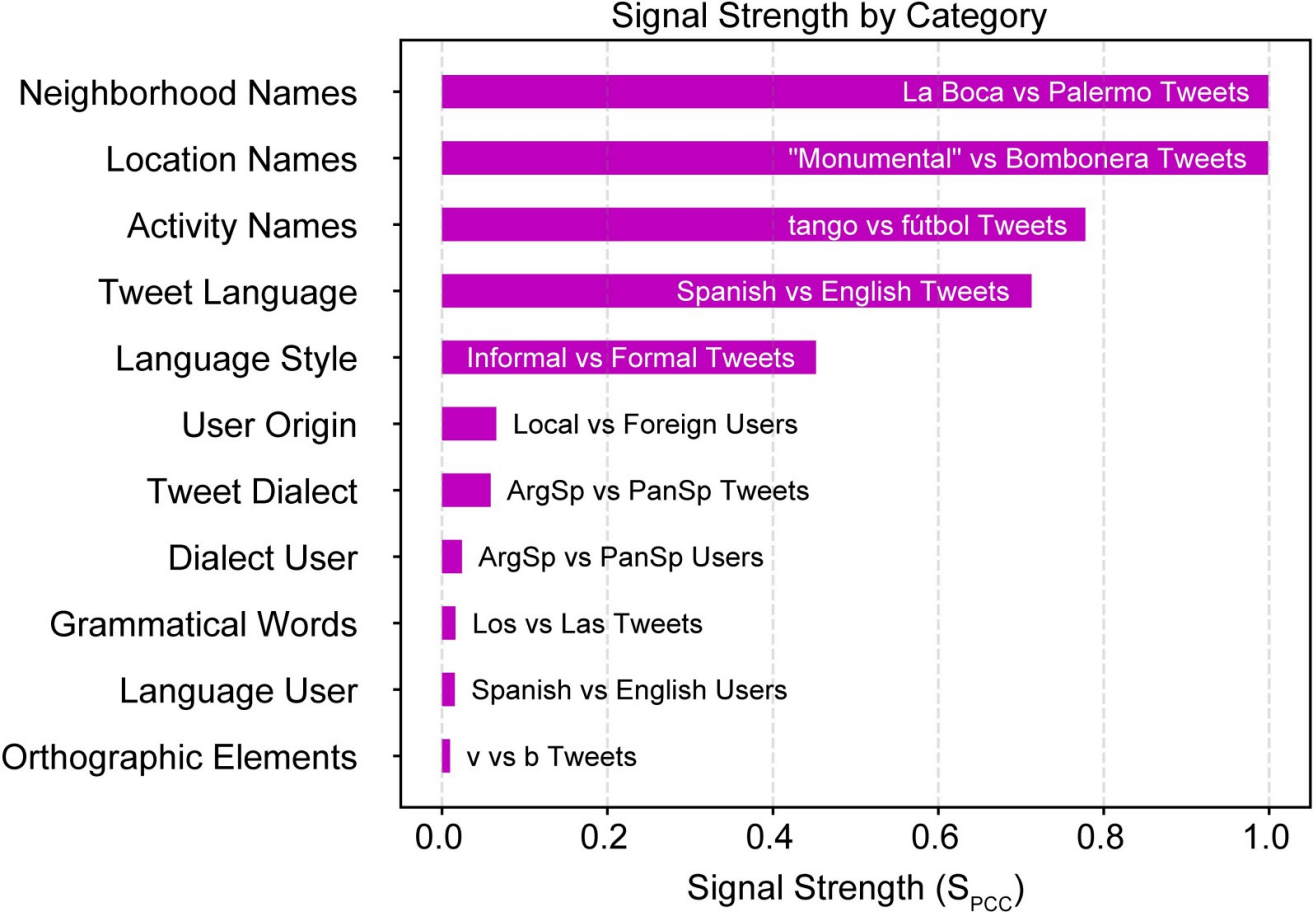
Bar chart of signal strength by category (case). Categories are arranged in order of decreasing signal strength (S_PCC_) from top to bottom. The first two cases, *Neighborhood Names* and *Location Names*, are expected to have very strong signal strength because the token sets directly encode location information. Similarly, *Orthographic Elements* and *Grammatical Words* are expected to have weak signals because the tokens have no direct connection to location. The cases with intermediate signal strength can be shown to have varying degrees of indirectness in their connection to location.

In the next analysis, we examine the connection between the signal strength and the category of sociolinguistic variation. A summary of the categories, ranked by signal strength, is plotted in Fig 15. The progression observed in this figure can be understood in terms of varying degrees of lexical and social connection of the defining tokens for each case to location. The lexical connection, which tends to be the stronger of the two, results from references to location intrinsic in the meaning of the tokens. The social connection, however, results from the behavior of people using the tokens, which is influenced by location, and is therefore less direct. In general, one can expect that the weaker the connection of the tokens to location is, the weaker the signal strength should be. For the cases of *Neighborhood Names* and *Location Names*, the tokens contain direct, lexical references to location, so it is not surprising that these categories should have very strong signals. On the other hand, the categories of *Orthographic Elements* and *Grammatical Words* do not have any lexical meaning, and therefore cannot encode a reference to location lexically, and also do not have strong social connections to location. The lack of any reference to location justifies their use as nulls for assessing noise levels, and also explains their very-low signal strengths. The intermediate-signal cases can be understood as having varying degrees of directness in the way they encode references to location. For example, in the case of *Activity Names*, the direct lexical reference of the tokens is to activities (i.e., tango and soccer), but there is an indirect reference to location via the venues where these activities are performed (e.g., tango bars and soccer fields). Since the reference is indirect, the strength of the signal is also less than for the direct-reference cases. Note also that while the *Neighborhood Names* and *Location Names* cases refer to individual locations, the *Activity Names* case refers (indirectly) to a category of locations. Since the reference is less unique (i.e., more distributed), the connection/signal strength associated with a particular location is naturally less. The *Language Style* case is unique among non-null cases in that there is a social connection to location but no lexical one. The presence of a robust signal therefore tells us that the effect of geosocial context on linguistic formality is quite strong.

For the cases *User Origin*, *Tweet Language*, *Tweet Dialect*, *Language User* and *Dialect User*, there is a linguistic connection to location via the definition of these categories. For instance, in the *User Origin* case, local users are defined as being from CABA, and for the *Dialect User* case, the ArgSp users are those that use an Argentinian dialect. However, the locations referenced in these cases represent large-scale regions such as countries and not urban-scale regions such as neighborhoods or particular location. The lack of reference to urban-scale locations therefore leads to weak lexical contributions to the signal strength. We can therefore assume that the signal strength for these cases is primarily social in origin. For example, the high signal strength for the *Tweet Language* case cannot be predicted based on the definition of this case. The strong signal therefore implies that the use of English is strongly driven by the geosocial context, which matches the conclusions drawn from the analysis for Fig 11 comparing this case to the *Language User* case. Comparing the user and tweet variants of the language and dialect cases (i.e., *Language User* vs *Tweet Language* and *Dialect User* vs *Tweet Dialect*), we see that in both cases the user variant had a weaker signal. This effect may have been due to the fact that for these user groups, there was substantial overlap between the target and reference groups, as measured by the large fraction of users that were “bilingual” and “bidialectal”, which reduced the degree of contrast between the groups.

### Comparison between statistical metrics

**Fig 16:**
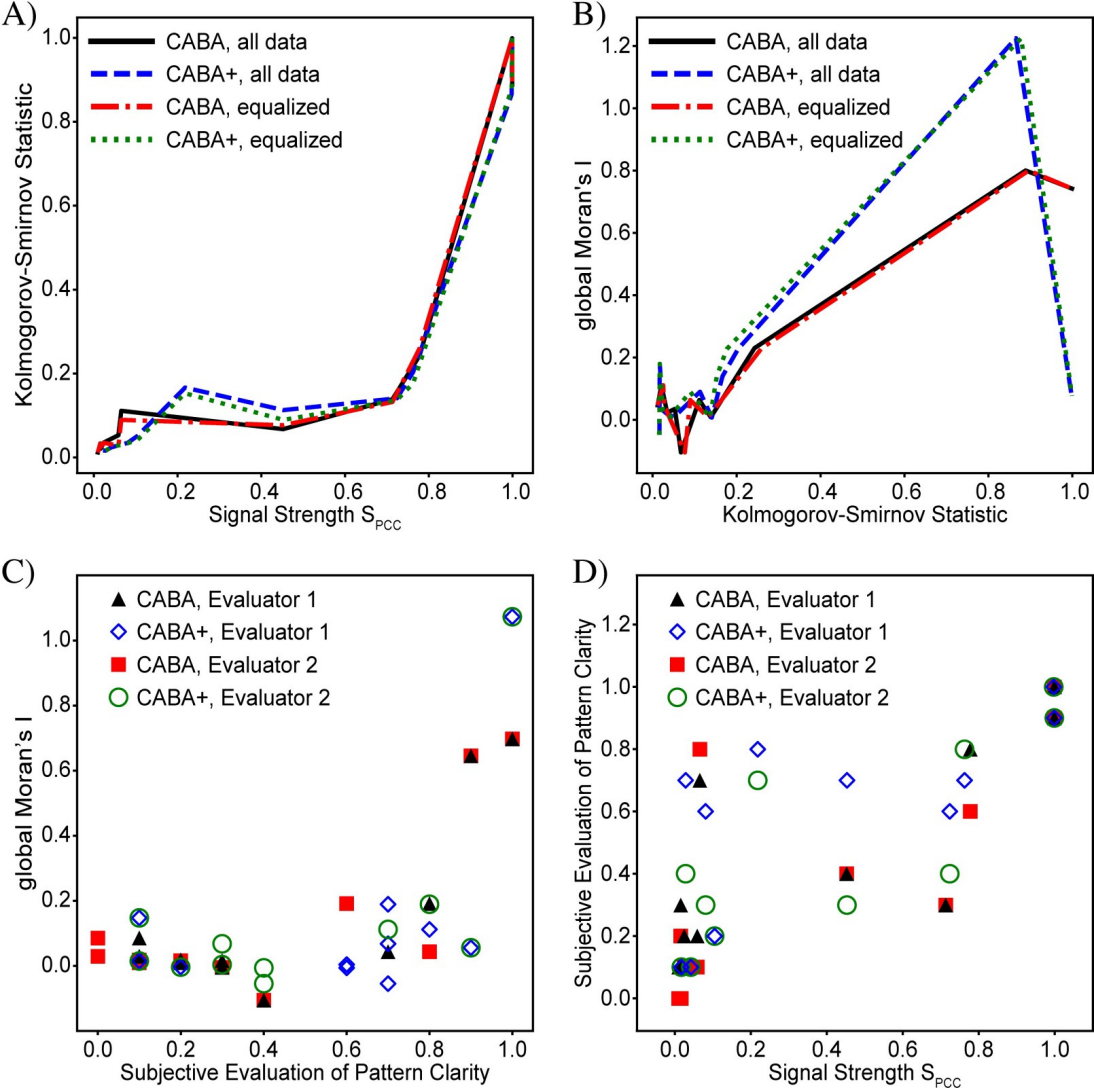
Comparison of statistical metrics. (A) Comparison of regression analysis with Kolmogorov-Smirnov analysis regarding presence of a signal. At low values and at high values, the two metrics compare very well to each other, but deviate noticeably for intermediate values. Based on visual inspection of the frequency-comparison plots in Fig 13 and the angle-histogram plots in Fig 14, the Pearson-correlation-coefficient-based metric appears to do a better job of quantifying the signal strength for the intermediate cases. Four plots are displayed. The black-solid and blue-dashed lines correspond to the data presented in the analyses above, for the CABA and CABA+ extents, respectively. The two curves show a similar but not identical behavior, with the biggest difference occurring for the local vs foreign user case (S_PCC_ = 0.22 vs 0.07 and KSS = 0.17 vs 0.09 for CABA+ and CABA, respectively). This discrepancy suggests that urban vs rural location has a greater impact on foreign users than downtown vs not-downtown within the city. The red-dot-dash and green-dotted curves are the equivalent of the previous two curves, except that the target and reference tweet counts have been equalized using randomized filtering of the larger set (see figures in S5 File). The equalized and non-equalized curves are nearly identical to each other, showing that the asymmetry in the number of tweets does not strongly affect the conclusions (B) Comparison of global Moran’s *I* vs the Kolmogorov-Smirnov statistic. In general, higher values of global Moran’s *I* are found for larger values of the Kolmogorov-Smirnov statistic, implying that (for these two metrics), clear patterns tend to go hand-in-hand with strong signals. This trend is broken for the last point corresponding to the *location names* case where the number of bins containing tweets was much lower than for any other case. This sparsity results in occupied bins tending to be disconnected, which is problematic for calculating global Moran’s *I* using nearest-neighbor weighting. (C) Comparison between global Moran’s *I* and a subjective evaluation of the pattern clarity. Both human evaluators independently judged the presence of a clear pattern on a scale from 0 to 1. The comparison shows first, that the two subjective judgements agree very well with each other, and second, that while global Moran’s *I* agrees with the subjective judgement at each end of the scale, it tends to undervalue the presence of a pattern relative to the subjective judgement for intermediate values. (D) Comparison of the subjective pattern evaluation vs the Pearson-correlation-coefficient-based signal strength metric. The plot shows (for these two metrics) that pattern clarity tends to correlate with signal strength, with especially good agreement at either end of the scale. As mentioned in the analyses above, however, there are also cases of clear patterns without strong signals and strong signals without clear patterns.

We have employed several statistical metrics to evaluate the language-use cases: the “signal strength” based on the Pearson correlation coefficient (S_PCC_), which targets the degree of correlation between target and reference tweet counts in each bin; the Kolmogorov-Smirnov statistic (KSS), which uses maximum differences in cumulative probability distribution to evaluate the likelihood that the spatial distributions of target and reference tweets are the same; and global Moran’s *I* (GMI), which uses global autocorrelation to quantify the degree of spatial clustering in the differential distribution maps. Since each of these metrics assess different aspects of the data, in this section we evaluate how these metrics compare to each other for the different language-use categories (Fig 16).

In principle, the KSS is analogous (but not equivalent) to our signal strength metric in that they both depend on the degree to which the social groups and geosocial contexts influence tweeting behavior. In order to apply the calculation of the KSS (which is inherently a comparison of 1-dimensional distributions) to our 2-dimensional differential distributions, we reshape our 2D distributions into 1D distributions by appending one row after the next. Fig 16A shows that, by in large, the greater the S_PCC_, the greater the KSS. The KSS, however, has a more nonlinear behavior, making it less sensitive to intermediate cases than the S_PCC_ metric. We therefore conclude that our regression analysis provides both a sensitive and robust metric for evaluating the strength of a variation signal.

We plot four variations of the graph corresponding to the two spatial extents (CABA+ and CABA) and to equalized and non-equalized versions of the data, where “equalized” refers to the total numbers of target and reference tweets. Since in several of the cases the target and reference tweet sets had a large disparity in numbers, we performed an additional analysis in which we randomly filtered tweets from the larger set, in order to equalize them, the purpose being to check for any influence of the tweet-count disparities. Fig 16A confirms that, in fact, the equalized and non-equalized versions of the analysis are nearly identical. In the supplementary information (S5 File), we also present differential distributions and frequency-comparison plots for the equalized versions of the cases with the largest disparities in target-reference tweet numbers (*Neighborhood Names*, *User Origin* and *Tweet Language*). These figures show that the relevant features are not substantially different. The results in Fig 16A also show mostly minor differences between the CABA+ and CABA analyses, with the largest difference occurring for the *local vs foreign user* case. Together with Fig 10A, which shows that on the CABA+ extent there are very few points favoring foreign users, this difference suggests that the urban/rural land-use distinction strongly impacts the location of foreigners.

We next evaluate the connection between the strength of the signal and the clarity of the pattern for our different cases. As discussed in the section on “Basic Approach”, the two properties are not intrinsically connected in that it is possible to have a strong signal without a clear pattern or a clear pattern without a strong signal. The strength of the signal relates to how strongly the social group or geosocial context determine the tweeting behavior, while the clarity of the pattern relates to how organized the regions of uniform geosocial context are. In principle and intuitively, however, one expects that relevant geosocial contexts *will* be spatially organized and that therefore the presence of a strong signal *should* correlate to the presence of a clear pattern. To test this assumption, we plot GMI (calculated using a weighting matrix that includes only nearest neighbors) against the KSS. The results show that for the cases chosen there is an overall correlation between the two metrics, confirming that, for the most part, cases with clearer patterns tend to have stronger signals. The plots show considerable differences between the equalized and full data sets for the two strongest-signal cases for which the majority of the bins had zero tweet counts. We attribute the discrepancy to the sparsity of occupied bins, which is problematic for the global Moran’s *I* statistic and which is worsened for the equalized data. This effect is also likely the reason why the case with the highest signal strength (*Location Names*) has very low values of GMI despite the fact that visually the patterns are exceedingly clear.

In order to get an independent assessment of the performance of the GMI metric, we cross-check the results against subjective evaluations of the pattern clarity performed by two human evaluators, asked to rank the eleven cases on a scale from 0 to 1 (Fig 16C). The results (see supplementary information S6 File) show that the GMI and subjective analyses agree for the clearest and least clear patterns, but that the GMI tends to underestimate the significance of the pattern relative to the subjective analysis for the intermediate cases. To understand the significance of this disagreement and isolate its origin requires a more dedicated analysis that will be the topic of future work. However, a likely ingredient is an imperfect tuning of the GMI calculation to account for case-specific issues such as high numbers of empty bins or characteristic distance scales over which the geosocial contexts exist.

As a final step, we compare the subjective pattern assessment to our metric for the signal strength (Fig 16D). This graph again shows agreement at either end of the scale, confirming the trend in Fig 16B that stronger signals are associated with clearer patterns. However, for intermediate values, the subjective assessments of pattern clarity showed both positive and negative disagreements relative to the signal strength, echoing the assertion that pattern clarity and signal strength are not intrinsically linked.

## Discussion and conclusion

In this section, we summarize the main points which were accomplished and then provide some perspective on what limitations were encountered and what opportunities exist for expanding on this work. It is evident that an important aspect of pursuing urban-scale analysis is to understand which geosocial contexts are likely to display a recognizable spatial pattern and which linguistic features can be associated with these contexts. These are empirical questions that depend on the social structure of a city and the people that live there, highlighting the necessity of exploring spatial patterns associated with different categories of linguistic features. To address this issue, we have used geolocated Twitter data to analyze eleven categories of language use with the objective of characterizing whether geographical patterns of linguistic variation driven by social groups and geosocial context can be resolved. By addressing a diversity of linguistic categories spanning from null cases where no variation is expected to strong cases where variation is anticipated, we provide a reference frame that enables ranking the degree of variation observed for different linguistic features. The agreement of these strong cases with expectations helps to validate the use of Twitter data as a valuable linguistic resource. We also expand on the scope of previous work in urban-scale linguistic variation by targeting linguistic features not connected to lexical meaning, such as language style and dialect.

Our analysis involved several steps: generation of detailed urban-scale maps of linguistic variation; development of a metric for evaluating the strength of the “signal”, (i.e., the degree of influence on linguistic behavior by social groups or geosocial context); assessment of the presence (i.e., “clarity”) of geographic patterns of linguistic variation; and, in most cases, identification of spatial patterns in geosocial context which plausibly explain the patterns of variation. To visualize the variations, we used the normalized difference of frequencies rather than the more typical *relative frequencies* as we found the former to be a more sensitive metric for our purposes. It should be emphasized, however, that a complete analysis benefits from the use of more than one metric, as each one tends to emphasize different aspects of the data. In order to validate our results, we compared our assessments of signal strength and pattern clarity with established metrics for evaluating the similarity of two distributions (i.e., the 2-sample Kolmogorov-Smirnov statistic) and the degree of spatial autocorrelation (i.e., global Moran’s *I*), respectively. We find that our assessments match well with the existing metrics for cases on either end of the signal-strength/pattern-clarity scales, but find that for intermediate cases our metrics provide evaluations that align more closely with intuitive assessments based on visual inspection of the data.

Analysis of specific cases have resulted in several important conclusions. First, we confirm the possibility of resolving urban-scale variations in linguistic features not associated with lexical meaning. In particular, we show that formal language has a higher probability of use in public than in private settings. Although this division aligns well with expectation [48], high resolution spatially-resolved information confirming this expectation is not trivial to obtain by other means. We also provide strong evidence that the PanSp dialect is well integrated into the social life of Buenos Aires and is therefore not solely a result of tourism or international business. This is the first quantitative evidence for the integration of PanSp in Buenos Aires and is thus a significant result for the sociolinguistic field.

Comparing cases against each other was also very fruitful. First, comparing the full set of language-use cases showed that the degree of measurable variation varied greatly with a trend that was expected. Not surprisingly, the strongest signals and clearest patterns were related to lexical categories with meanings directly encoding reference to a location, while the weakest signals and least clear patterns were associated with grammatical cases that generally do not encode location. Second, comparing categories associated with geosocial contexts with those associated with social groups, we found that signals were generally stronger for the former than for latter. For the Language User and Dialect User cases, this was likely attributable to the large overlap between the target and reference user groups, leading to a lack of contrast. Nevertheless, we do find distinctive patterns associated with users, such as the concentration of PanSp dialect users on the outskirts of CABA+, the preference for English users in the North, and a clear localization of foreign users in the city center. Third, by comparing cases analyzing where a particular user-group pair (e.g., Spanish vs English users and ArgSp vs PanSp users) tweeted in general with cases analyzing where the same user-group pair tweeted a particular tweet variant, we demonstrated that it is possible to directly and unambiguously measure the influence of geosocial context on tweeting behavior. Finally, we show that the presence of a clear spatial pattern of variation is *not* synonymous with having a strong variation signal. This point is illustrated by the user case of Locals vs. Foreigners which exhibits a clear spatial pattern but one of the lowest measures of signal variation.

The results summarized above represent only the outline of a methodology involving several ways of using social-media data to analyze the connections between language use and geosocial context on an urban scale. It is clear that many further developments are required to make the most of this rich data source. In particular, optimizing extraction of tweets is a key issue in order to provide the quality and quantity of data required for some analyses. The use of token sets to select tweets, while enabling highly precise categorizations, has several limitations in this regard. First, this approach can be inefficient, with the tweets selected for each contrasting set in some cases representing 1% or less of the total corpus. Second, finding pairs of token sets that are truly contrastive in the desired sense is time consuming and suffers from ambiguities associated with the multiple meanings of words. Machine-learning methods offer promising alternatives for selecting tweets. Neural language models such as BERT [49,50], for example, may provide solutions to the ambiguity problem by using linguistic context information [50]. However, these models, while very powerful, have so far only been applied to a limited number of linguistic categories.

A second area requiring work is the quantification of the pattern clarity. Here we have applied global Moran’s *I* using nearest-neighbor weighting as a measure of the overall degree of clustering within the pattern. While this approach provided results which were coarsely in line with subjective assessment of the patterns, it is clear that a rigorous treatment of individual cases requires an analysis which is more tailored. This necessity is highlighted by the case of *location names*, where the small number of bins containing tweets led to spurious results with the global Moran’s *I* metric. This case also highlights a broader issue which is the size of a particular geosocial context, which we have, so far, not addressed. By “size”, we refer to the spatial extent over which the geosocial context can be considered uniform. The presence of a pattern, whether ascertained by subjective or quantitative means, is connected to the degree of spatial clustering. Evidently, if the size of a particular context is on the same scale as or smaller than the size of a bin, then no patterns will be detected. This circumstance explains how it is possible to have a strong signal without a clear pattern. In the *location names* case, the locations referred to were two soccer stadiums, and the data shows that the tweets were highly localized to the stadiums themselves and one or two adjacent bins. In this case, the geosocial context is essentially restricted to the stadium itself and its immediate surroundings. For the CABA+ analysis, the tweets referring to the stadium *“Monumental” Antonio Vespucio Liberti* occupied a single bin. One can imagine, however, that given sufficient data and geolocation accuracy, using bins of size significantly smaller than the size of the stadium should allow visualization of the clustering associated with this context. The implication is that the resolution of the binning and the size of the weighting region for the autocorrelation analysis should be adapted to the size of the geosocial context. In addition, as the degree of clustering can be expected to vary within the spatial extent of the analysis, using a *local* metric of autocorrelation should provide a clearer assessment of the features of the differential distributions to compare with spatial maps of geosocial context, and is therefore essential for a complete analysis. A complementary aspect of this work is development of a set of geosocial-context maps (e.g., Fig 11G) with content that is richer, more informative and captures the complex structure of cities to use for comparison with the differential distributions. In the current digital age, many opportunities exist for collecting this information. Natural tools for this effort are Geographical Information Systems (GIS) [51] which enable characterization of the social function of buildings and other location types within a city [52].

Many questions remain to be answered, including: how representative is Twitter data of a particular social group or social circumstance, and to what extent are the dependence we observe of pattern clarity and signal strength on linguistic category generalizable? Our work presents analysis of geographical variations in categories of language use that open up new possibilities for studying the complex relationship between language and space, especially on urban scales. We provide evidence that these variations are driven by geosocial context and by social groups and lay the groundwork for further studies that explore these relationships.

## Supporting information

S1 File Full_list_of_Spanish_Tweet_IDs. (7Z)

S2 File Full_list_of_English_Tweet_IDs. (7Z)

S3 File Tweet Specifier Lists. (DOCX)

S4 File Exact CABA_Statistics. (XLSX)

S5 File Equalized versions of asymmetric cases. (DOCX)

S6 File Subjective assessment of spatial patterns. (XLSX)
