# Supplementary material for: Geolocation of multiple sociolinguistic markers in Buenos Aires
Source: PLoS One. 2022 Sep 9;17(9):e0274114. doi: 10.1371/journal.pone.0274114 (PMC9462814; doi:10.1371/journal.pone.0274114)
Supplement: S3 File — (DOCX) [file pone.0274114.s003.docx]

**S3. Tweet Specifier Lists**

| Category | Description | Selection Mode | Specifiers |
| --- | --- | --- | --- |
| Orthographic Elements | v vs b Tweets | token list | **Specification:** Collect all tweets containing tokens from the target and reference lists.  **Target Tokens** = ["v"]  **Reference Tokens** = ["b"] |
| Grammatical Words | Los vs Las Tweets | token list | **Specification:** Collect all tweets containing tokens from the target and reference lists.  **Target Tokens** = [" los "]  **Reference Tokens** = [" las "] |
| Neighborhood Names | La Boca vs Palermo Tweets | token list | **Specification:** Collect all tweets containing tokens from the target and reference lists.  **Target Tokens** = ["a Boca "]  **Reference Tokens** = [“ Palermo “] |
| Location Names | “Monumental” vs Bombonera Tweets | token list | **Specification:** Collect all tweets containing tokens from the target and reference lists.  **Target Tokens** = ['en Estadio "Monumental" Antonio']  **Reference Tokens** = ["Bombonera"] |
| Activity Names | tango vs fútbol Tweets | token list | **Specification:** Collect all tweets containing tokens from the target and reference lists.  **Target Tokens** = ["tango ", "Tango "]  **Reference Tokens** = ["fútbol ", "Fútbol "] |
| Tweet Language | Spanish vs English Tweets | metadata: ‘lang’ | **Specification:** Collect all tweets associated with target and reference languages.  **Target (Spanish) Language** = ‘es’  **Reference (English) Language** = ‘en’ |
| Language User | Spanish vs English Users | metadata: ‘lang’ + metadata: ‘user ID’ | **Specification:** Collect all tweets containing tokens from the target and reference lists.  **Target (Spanish) Language** = ‘es’  **Reference (English) Language** = ‘en’ |
| Language Style | Informal vs Formal Tweets | token list | **Specification:** Collect all tweets containing tokens from the target and reference lists.  **Target (Informal) Tokens =** [" feca ", "quilombo", "piola", "cagatearse", "cagatina", "cagar", "boluda", " boludo", " Bolud", " culo ", "puta", "mierda"]  **Reference (Formal) Tokens =** ["café", "escándalo", "comprensiva", "sentir miedo", "Diarrea", "perjudicar", "estúpid", "por favor ", " señor", "Señor", " trasero ", "prostituta", "excremento"] |
| Tweet Dialect | ArgSp vs PanSp Tweets | token list | **Specification:** Collect all tweets containing tokens from the target and reference lists.  **Target (ArgSp) Tokens =** ["Seguínos ", "Vení ", "Che,", " che," "Chau", " chau", "Bolud", "boludo", "boluda", " vos ", "Vos ", ' sos ', ' sentís ', ' sentis ', ' decís ', ' decis ', ' seguís ', ' seguis ', ' venís ', 'venis', ' vivís ', ' vivis ', ' salís ',' salis ', ' elegís ', ' elegis ', ' pedís ',' pedis ', ' referís ', ' referis ', ' preferís ', ' preferis ', ' escribís ', ' escribis ', ' subís ', ' subis ', ' recibís ', ' recibis ', ' conseguís ', ' conseguis ', ' abrís ', ' abris ', ' sufrís ', ' sufris ', ' decidís ', 'decidis', ' morís ', ' compartís ', 'compartis', ' definís ', 'definis', ' dormís ', ' dormis ',' coincidís ', ' coincidis ', ' convertís ', ' convertis ',' servís ', ' servis ', ' arrepentís ', ' arrepentis ', ' reís ', ' reis ', ' descubrís ', ' descubris ', ' consumís ', ' consumis ', ' existís ', 'existis ',' permitís ', ' permitis ', ' competís ', ' cometis ', ' dirigís ', ' percibís ', ' atribuís ', ' adherís ', ' vestís ', ' repetís ', ' medís ', ' asumís ', ' construís ', ' convivís ', ' divertís ', ' invertís ', ' producís ', ' describís ', ' sonreís ', ' transmitís ', ' aburrís ', ' conducís ', ' insistís ', ' rendís ', ' mentís ', ' cubrís ', ' adquirís ', ' incluís ', ' sugerís ', ' suscribís ', ' confundís ', ' exigís ', ' advertís ', ' unís ', ' recurrís ', ' dividís ', ' resistís ', ' reunís ', ' combatís ', ' emitís ', ' discutís ', ' fundís ', ' oís ', ' concebís ', ' reducís ', ' contribuís ', ' hundís ', ' inscribís ', ' nutrís ', ' prohibís ', ' sobrevivís ', ' perseguís ',' deprimís ', ' despedís ', ' aplaudís ', ' introducís ', ' corregís ', ' admitís ', ' hervís ', ' asistís ', ' aludís ', ' desmentís ', ' destruís ', ' ingerís ', ' sumergís ', ' repartís ', ' traducís ', ' transferís ', ' teñís ', ' escupís ', ' intuís ', ' acudís ', ' distribuís ', ' disminuís ', ' contradecís ', ' intervenís ', ' pulís ', ' fingís ', ' residís ', ' evadís ', ' difundís ', ' añadís ', ' curtís ', ' interrumpís ', ' revivís ', ' sacudís ', ' reprimís ', ' requerís ', ' exhibís ', ' concluís ', ' exprimís ', ' imprimís ', ' infringís ', ' herís ', ' huís ', ' presidís ', ' omitís ', ' reproducís ', ' remitís ', ' sustituís ', ' restringís ', ' resumís ', ' seducís ', ' sobresalís ', ' prevenís ', ' impartís ', ' incurrís ', ' influís ', ' concurrís ', ' comprimís ', ' aducís ', ' derretís ', ' eludís ', ' freís ', ' expandís ', ' excluís ', ' escurrís ', ' esgrimís ', ' redescubrís ', ' prostituís ', ' provenís ', ' prescindís ', ' oprimís ', ' irrumpís ', ' maldecís ', ' revertís ', ' rescindís ', ' resentís ', ' transcribís ', ' transgredís ', ' ungís ', ' zambullís ', ' reincidís ', ' reñís ', ' suprimís ', ' sucumbís ', ' invadís ', ' predecís ', ' presumís ', ' proferís ', ' esparcís ', ' esculpís ', ' eximís ', ' impedís ', ' digerís ', ' desvivís ', ' devenís ', ' desdecís ', ' desistís ', ' deducís ', ' asentís ', ' afligís ', ' adscribís ', ' ceñís ', ' aturdís ', ' consentís ', ' constituís ', ' reís ', ' subís ', ' tenés ', ' podés ', ' querés ', ' entendés ', ' mantenés ', ' pensás ', ' empezás ',' mantenés ', ' andá ']  **Reference (PanSp) Tokens =** ["Sigue ", "Ven ", "asta luego", "Oye", " tú ", "Tú ", ' eres ', ' sientes ', ' dices ', ' sigues ', ' vienes ', ' vives ', ' sales ', ' eliges ', ' pides ', ' refieres ', ' prefieres ', ' escribes ', ' subes ', ' recibes ', ' consigues ', ' abres ', ' sufres ', ' decides ', ' mueres ', ' compartes ', ' defines ', ' duermes ', ' coincides ', ' conviertes ', ' sirves ', ' arrepientes ', ' ríes ', ' descubres ', ' consumes ', ' existes ', ' permites ', ' compites ', ' diriges ', ' percibes ', ' atribues ', ' adhieres ', ' vistes ', ' repites ', ' mides ', ' asumes ', ' construes ', ' convives ', ' diviertes ', ' inviertes ', ' produces ', ' descubres ', ' sonríes ', ' transmites ', ' aburres ', ' conduces ', ' insistes ', ' rindes ', ' mientes ', ' cubres ', ' adquieres ', ' inclues ', ' sugieres ', ' suscribes ', ' confundes ', ' exiges ', ' adviertes ', ' unes ', ' recurres ', ' divides ', ' resistes ', ' reunes ', ' combates ', ' emites ', ' discutes ', ' fundes ', ' oyes ', ' concibes ', ' reduces ', ' contribuyes ', ' hundes ', ' inscribes ', ' nutres ', ' prohibes ', ' sobrevives ', ' persigues ', ' deprimes ', ' despides ', ' aplaudes ', ' introduces ', ' corrigues ', ' admites ', ' hierves ', ' asistes ', ' aludes ', ' desmientes ', ' destruyes ', ' ingieres ', ' sumerges ', ' repartes ', ' traduces ', ' transfieres ', ' tiñes ', ' escupes ', ' intuyes ', ' acudes ', ' distribues ', ' disminues ', ' contradices ', ' intervienes ', ' pules ', ' finges ', ' resides ', ' evades ', ' difundes ', ' añades ', ' curtes ', ' interrumpes ', ' revives ', ' sacudes ', ' reprimes ', ' requieres ', ' exhibes ', ' concluyes ', ' exprimes ', ' imprimes ', ' infringes ', ' hieres ', ' huyes ', ' presides ', ' omites ', ' reproduces ', ' remites ', ' sustituyes ', ' restringes ', ' resumes ', ' seduces ', ' sobresales ', ' previenes ', ' impartes ', ' incurres ', ' influyes ', ' concurres ', ' comprimes ', ' aduces ', ' derrites ', ' eludes ', ' fríes ', ' expandes ', ' excludes ', ' escurres ', ' esgrimes ', ' redescubres ', ' prostituyes ', ' provienes ', ' prescindes ', ' oprimes ', ' irrumpes ', ' maldices ', ' reviertes ', ' rescindes ', ' resientes ', ' transcribes ', ' unges ', ' zambulles ', ' reincides ', ' reñes ', ' suprimes ', ' sucumbes ', ' invades ', ' predices ', ' presumes ', ' profieres ', ' esparces ', ' esculpes ', ' eximes ', ' impides ', ' digieres ', ' desvives ', ' devienes ', ' desdices ', ' desistes ', ' deduces ', ' asientes ', ' afliges ', ' adscribes ', ' aturdes ', ' consientes ', ' constituyes ', ' ríes ', ' subes ', ' tienes ', ' puedes ', ' quieres ', ' entiendes ', ' piensas ', ' empiezas ', ' mantienes '] |
| Dialect User | ArgSp vs PanSp Users | token list + metadata: ‘user ID’ | **Specification:** Collect all tweets with user ID associated with tweets containing tokens from the target and reference lists.  **Target (ArgSp) Tokens** = same as above  **Reference (PanSp) Tokens** = same as above |
| User Origin | Local vs Foreign Users | metadata: ‘location’ | **Specification:** Collect all tweets from users have the target and reference locations.  **Target (Local) Locations** = ['Buenos Aires, Argentina', 'Ciudad Autónoma de Buenos Aire', 'Buenos Aires', 'Ciudad Autónoma de Buenos Aires, Argentina', 'La Plata, Argentina', 'AMBA', 'Buenos Aires - Argentina', 'Quilmes, Argentina', 'Ciudad del Libertador General', 'La Plata', 'Tigre, Argentina', 'Capital Federal', 'Buenos Aires, Villa Ballester', 'BUENOS AIRES', 'Lomas de Zamora, Argentina', 'buenos aires', 'San Isidro, Argentina', 'Buenos Aires Argentina', 'Vicente López, Argentina', 'Moreno, Argentina', 'la plata', 'Merlo, Argentina', 'Pilar, Argentina', 'Lanús Oeste, Argentina', 'Caballito, buenos aires', 'buenos aires ,argentina', 'Morón, Argentina', 'Avellaneda, Argentina', 'CABA', 'Hurlingham, Argentina', 'San Miguel, Argentina', 'Berazategui, Argentina', 'Argentina, Mataderos', 'Buenos Aires, Argentina.', 'Quilmes', 'Almirante Brown, Argentina', 'Argentina, Buenos Aires', 'Ciudad Autónoma de Buenos Aires', 'Lanus', 'villa crespo argentina', 'San Justo, Argentina', 'Belén de Escobar, Argentina', 'Buenos Aires,Argentina', 'Buenos Aires', 'Monte Grande, Argentina', 'Ciudad de Buenos Aires', 'Buenos Aires. Argentina', 'ciudad de buenos aires', 'Florencio Varela, Argentina', 'Palermo', 'Don Torcuato,Buenos Aires', 'Argentina - Buenos Aires', 'San Fernando, Argentina', 'Buenos Aires, Argentina', 'Marcos Paz, Argentina', 'Victoria, Buenos Aires', 'San Vicente, Argentina', 'Hurlingham', 'Buenos aires', 'Buenos Aires-Argentina', 'San Martín, Argentina', 'La Boca', 'Lanús', 'Ituzaingó Centro, Argentina', 'Boedo, Buenos Aires, Argentina', 'ARGENTINA AVELLANEDA', 'Lomas de Zamora', "Av.Balbín 1044.L.6 / D'elia 1409 entre Charlone y Belgrano. San Miguel.", 'Caballito', 'buenos aires, argentina', 'Ranelagh-Buenos aires', 'Castelar', 'Berazategui', 'Perdido en General San Martín', 'Parque Chacabuco', 'Buenos Aires-Argentina-', 'Acevedo 350 villa crespo', 'Belgrano, Capital Federal', 'Buenos Aires, ARGENTINA', 'José María Ezeiza, Argentina', '-34.652832,-58.595634', 'Ciudad de Buenos Aires, AR', 'Wilde', 'rafael calzada , buenos aires', 'ezeiza', 'Ramos Mejia', 'Bs As', 'Pilar', 'La Plata, Buenos Aires', 'Buenos Aires.', 'Wilde City...', 'Ensenada, Argentina', 'Buenos Aires - Argentina', 'Capital Federal, Buenos Aires', 'Caseros, Argentina', 'Republica de Mataderos', 'Bs As Argentina', 'Banfield', 'Bernal Centro BS AS', 'Rafael Calzada', 'buenos aires', 'Remedios de Escalada', 'Buenos Aires , Argentina', 'Tigre', 'Caballito, Argentina.', 'C.A.B.A', 'Capital Federal - Argentina', 'Berisso, Argentina', 'Rodriguez Peña 48, C.A.B.A.', 'BUENOS AIRES, ARGENTINA', 'BUENOS AIRES ° ARGENTINA', 'GUERNICA', 'CABA, Argentina', 'Buenos Aires, CABA, Argentina', 'Cdad. Madero, BA., Argentina', 'Don Torcuato, Buenos Aires', 'Florencio Varela', 'buenos aires argentina', 'Patricios/Bs.As./ARGENTINA', 'Los Polvorines, Argentina', 'Buenos Aires- Argentina', 'Bs.As. Argentina', 'Marcos Paz, Buenos Aires', 'Ramos Mejia, Buenos Aires', 'General Rodríguez, Argentina', 'lomas', 'La Paternal', 'floresta', 'Buenos Aires City Region', 'Merlo, Buenos Aires, Argentina', 'Buenos Aires. Argentina.', 'Saavedra', 'Argentina. Caballito', 'Avellaneda', 'Burzaco, Buenos Aires', 'C.A.B.A. BS.AS. RCA. ARGENTINA', 'Monte Grande', 'Mariano Acosta, Buenos Aires', 'Parque Patricios', 'marcos paz bs as argentina', 'Jose Marmol, Argentina', 'LANUS', 'General San Martín, Argentina', 'San Miguel', 'Mariano Acosta', 'buenos aires - ARGENTINA', 'Temperley', 'Puerto madero ARG.', 'Mataderos', 'Tribunales - Capital Federal', 'La plata', 'Matheu', 'Lanús, Argentina', 'Guernica, Argentina', 'Olivos', 'Bs.As.- Argentina', 'Villa Crespo pero de Quilmes', 'Floresta', 'Ciudadela', 'Boedo', 'Haedo', 'Bs. As. Argentina', 'Buenos Aires ,Argentina', 'Pilar, Buenos Aires, Argentina', 'Botánico Sur, CABA', 'Lanus, Buenos Aires, Argentina', 'ITUZAINGO ZONA OESTE BS.AS.', 'Quilmes. Argentina', 'Gorriti 4802 Palermo Soho', 'Liniers', 'Villa del Parque', 'Villa Urquiza', 'Lomas del Mirador', 'Ciudad de Bs. As. Argentina', 'Morón, Buenos Aires, Argentina', 'Villa Lugano, Argentina', 'Barracas', 'La Plata, Buenos Aires', 'San Isidro', 'San Isidro, Buenos aires', 'Bernal -Bs As-Argentina', 'Capital Federal, Argentina', 'Rio de la Plata', 'C. A. de Buenos Aires', 'Monserrat , CABA , Argentina', 'Malvinas Argentinas.', 'Merlo, Buenos Aires', 'Wilde', 'Villa Pueyrredón', 'capital federal argentina', 'BA - Argentina', 'Sarandi, Avellaneda', 'Villa Soldati, Argentina', 'BS.AS.ARGENTINA', 'Buenos Aires - Argentina', 'Pablo Nogues', 'Malabia 1491 Buenos Aires - Ar', 'Belén de Escobar', 'Moreno, Buenos Aires', 'Argentina / Bs As.', 'Bs. As.', 'lomas de zamora', 'Avellaneda, Buenos Aires', '9 de julio 1128, Lanus Este.', 'Rincon de Milberg, Tigre', 'Tigre, Buenos Aires', 'Buenos Aires City Region, Argentina', 'Ensenada', 'C.A.B.A.', 'Burzaco', 'Bs.as. san isidro-la cava', 'Villa Ballester', 'Ituzaingo', 'La Plata-Bs As-Argentina', 'La Plata-Tolosa', 'Argentina buenos aires', 'Tapiales, Buenos Aires', 'San Fernando', 'San Justo', 'quilmes', 'san miguel', 'San Pedro, Argentina', 'Pilar, Buenos Aires', 'Ezeiza', 'Hudson,Buenos Aires, Argentina', 'Canning, Ezeiza', 'Merlo, Bs. As .', 'Santa Fe 836, Morón, Argentina', 'rugby//BsAs Argentina', 'San Fernando, GBA norte', 'Merlo', 'munro buenos aires argentina', 'San Vicente', 'Belgrano, CABA. Argentina', 'cap.fed', 'Buenos Aires, AR', 'san isidro', 'Villa Bosch', 'Buenos Aires - Argentina.', 'La Plata', 'Quilmes, Buenos Aires', 'CAPITAL FEDERAL', 'BUENOS AIRES- LA MATANZA', 'Don Torcuato', 'Buenos aires - Argentina', 'san miguel.buenos aires', 'Tigre, Buenos Aires, Argentina', 'Lugano', 'González Catán, Argentina', 'Palermo, CABA, Argentina', 'Argentina. Mataderos Cap.Fed !', 'longchamps Argentina bs Aires', 'Caballito', 'CABALLITO- C.A.B.A 4903-7981', '9 y 10 Junio 2018 Bs. As.', 'San Martin', 'Santos lugares', 'Bs As, Argentina', 'Barracas, CABA', 'Lanus Buenos Aires', 'lomas del mirador, argentina', 'Villa Crespo', 'Palermo Buenos Aires', 'Argentina Buenos Aires', 'Isidro Casanova', 'Argentina - Buenos Aires- Cap', 'Berazategui, Buenos Aires', 'buenos aires - argentina', 'Munro', 'C.A.B.A Argentina', 'Jose Marmol 1808-San justo', 'Palermo, Capital Federal', 'MATADEROS', 'Bs.As.', 'Caseros', 'haedo bsas', 'Valentin Alsina, Lanus Oeste', 'Berisso', 'Cap Federal', 'Buenos aires, Argentina', 'buenos aires-argentina', 'Caseros, Buenos Aires.', 'La Plata, Bs As, Argentina', 'Villa Lugano', 'Lanús Oeste', 'Temperley', 'Buenos Aires ARGENTINA', 'Argentina, BAires, Banfield', 'Ciudad Autónoma Buenos Aires', 'Buenos Aires, Arg', 'Capital federal', 'Lanus', 'Andres Baranda 941- Quilmes', 'La Plata,Buenos Aires,', 'Facultad de Medicina - U.B.A. - Argentina', 'Buenos Aires 🇦🇷 Argentina', 'Republica de la Boca', 'Villa Luro', 'Castelar, Buenos Aires', 'Almagro, Buenos Aires', 'capital federal', 'Argentina, Capital Federal', 'Buenos Aires, Capital Federal', 'Aime Paine 1607 Puerto Madero', 'avellaneda argentina', 'platense en lanus', 'Lanus oeste', 'Fotógrafo, Buenos Aires', 'Wilde, Buenos Aires, Argentina', 'Lanús Este, Argentina', 'Ituzaingo Bs.As Argentina', 'banfield', 'Loma Hermosa', 'CABA - Argentina', 'Luzuriaga', 'Buenos Aires, Argentina Moreno', 'lanus', 'Ituzaingó', 'Argentina - Bs.As - Lanus Este', '', 'Moreno', 'Buenos Aires, ARG', 'V.Urquiza', 'Trenque lauquen - Bs. As.', 'Gerli', 'Ciudad Autónoma Buenos Aires.', 'Martinez, San Isidro', 'Capital Federal, Argentina', 'Salta 1913, Lanus Este', 'Buenos Aires; Argentina', 'AVELLANEDA', 'Grecia 3401 Nuñez 47018920', 'san fernando, buenos aires', 'República de Mataderos', 'Villa Madero', 'C.A.B.A, Palermo', 'Lanus Este', 'ASUNCION 4168Devoto.', 'Bernal', 'Belgrano', 'Morón', 'Berazategui, B.A.', 'Ramos Mejia Buenos Aires', 'Lomas de Zamora, Buenos Aires', 'La Plata Buenos Aires', 'Argentina (Buenos Aires)', 'Bs.As', 'Argentina.Buenos Aires.', 'FLORESTA', 'CABA-Argentina', 'Caba', 'Rawson 3719 La Lucila Bs As', 'Ramos Mejia \| Buenos Aires', 'Merlo,Buenos Aires', 'el palomar', 'berazategui', 'C.A. Buenos Aires', 'Berazategui, Bs As - Argentina', 'ituzaingo', 'La Boca - Argentina', 'San Isidro, Buenos Aires', 'Quilmes - Bs As - Argentina', 'Buenos aires, Argentina :)', 'iPhone: -34.618237,-58.451675', 'General belgrano , Buenos As', 'Buenos Aires y viceversa', 'Buenos Aires, Argentina 🇦🇷', 'capital federal.', 'La Plata, Capital Pcia Bs. As.', 'Pilar Bs. As.', 'BUENOS AIRES - ARGENTINA', 'BA', 'ÜT: -34.6223036,-58.3932047', 'bs as', 'BUENOS AIRES ARGENTINA', 'Argentina - Buenos Aires', 'bs as argentina', 'iPhone: -34.609413,-58.392982', 'Caballito, Buenos Aires', 'Argentina,Buenos Aires', 'caballito', 'lanus buenos aires', 'Kilmes, Bs As', 'Argentina, Buenos Aires.', 'Buenos Aires (Capital)', 'San Miguel Bs. As.', 'Victoria buenos aires', 'Palermo caba', 'Berazategui', 'bs as la mejor ciudad mundial', 'Belgrano R, BA', 'Palermo, Argentina', 'Bernal ( Quilmes)', 'Mi Buenos Aires querido.', 'La Plata Buenos Aires Argentin', 'La Plata (Arg)', 'Buenos Aires, Argentina -', 'Palermo', 'Belgrano', 'Quilmes', 'moreno, buenos aires', 'Martínez, Buenos Aires', 'Ituzaingo, Buenos Aires', 'Buenos aires, argentina', 'La plata', 'Ezeiza buenos aires', 'Belgrano. Caba. Arg.', 'Liniers, Buenos Aires', 'Capital Federal Buenos Aires', 'CABA, ARGENTINA', 'Pilar Bs As Argentina', 'BUENOS AIRES / ARGENTINA!!!!!', 'La Plata. Buenos Aires', 'Cementerio de Recoleta', 'BS.AS ARGENTINA (GLEW)', 'Villa Urquiza, BA', 'ÜT: -34.551298,-58.465947', 'cap fed', 'Saavedra,Bs. As. Argentina', 'República de la Boca Argentina', 'Sarmiento 1983, CABA', 'Flores CABA', 'buenos aires, Argentina', 'Villa del parque,bs as,argentina!!!', 'Haedo , Bs As , Argentina', 'Bella Vista, AR', 'Buenos Aires - Argentina', 'Lanús, Buenos Aires, Argentina', 'Arg, Bs As', 'Ciudad de Buenos AIres', 'Argentina, Buenos aires', 'argentina bs as. cap fed', 'San Telmo', 'Bs As', 'BA, Argentina', 'Ciudad Evita Buenos Aires', 'Av. Cabildo 1156 1A /4788-2992', '♡Buenos Aires,Argentina♡', 'villa crespo argentina', 'Palermo - Buenos Aires - Arg.', 'Merlo, Bs As, Argentina', 'Monumental', 'Villa Del Parque', 'San Telmo, Buenos Aires', 'La Boca Argentina', 'caba', 'Bs As - Argentina', 'Quilmes, Argentina.', 'Av 7 1130 E/ 55 y 56 la plata', 'Bs as, Argentina', 'Las Cañitas,C.A.B.A, Argentina', 'Monte Grande Buenos Aires', 'Bajo Belgrano - C.A.B.A.', 'Buenos Aires-Argentina Revival', 'Munro Buenos Aires Argentina', 'Argentina- Capital Federal', 'VILLA DE MAYO, BUENOS AIRES', 'Quilmes, Argentina📱1149692972', 'Cidade Autónoma de Buenos Aires, Argentina', 'Capital Feferal, buenos Aires', 'Benavidez, tigre - buenos aires', 'Buenos AIres', 'Bs.As Argentina Tablada.', 'Argentina -Ciudad Evita', 'Buenos Aires • Argentina', 'Av cabildo 2200 (Belgrano)', 'CABA - Buenos Aires', 'Buenos aires', 'Microcentro, CABA, Argentina', 'Congreso, Capital Federal', 'Argentina (Buenos Aires, Santa Fe y Neuquén), Chile (Santiago)', 'Quilmes ★ 4253-6835', 'C.A.B.A. Buenos Aires', 'LA PLATA', 'General Las Heras Buenos Aires', 'Cdad. Autónoma de Buenos Aires', 'Buenos aires Argentina', 'Martinez, Buenos Aires', 'José C. Paz, Buenos Aires, Argentina.', 'Buenos Aires, Argentina.', 'Avellaneda. Buenos Aires', 'San Miguel, Buenos Aires', 'R. Peña 336 Buenos Aires', 'Flores Bs As Argentina', 'Bs As - Argentina', 'Bayres, Argentina', 'República de la Boca', 'Argentina, Bs As', 'Caballito, Capital Federal', 'Buenos Aires, Castelar', 'San Martín, Buenos Aires', 'Argentina Buenos Aires.', 'Villa del Parque CABA', 'Tres de Febrero, Buenos Aires', 'Buenos Aires (Argentina)', 'Argentina, buenos aires', 'ÜT: -34.9045111,-57.9776751', 'C.a.ba', 'Buenos Aires Argentina', 'Buenos Aires City Region, Arge', 'Bs as, Argentina', 'Quilmes - Buenos Aires', 'Merlo Buenos Aires Argentina', 'Bs. As. Argentina', 'buenos aires - Argentina', 'La Reja Bs As Argentina', 'San Martin, Buenos Aires', 'Castelar Sur, Buenos Aires', 'Colegiales. Caba. Arg.', 'CABA BUENOS AIRES ARGENTINA', 'Buenos Aires , Argentina.', 'Villa luro', 'Santos Dumont 3528 Colegiales ☎4553-1639', 'QUILMES, ARGENTINA', 'La Boca, CABA, ARGENTINA', 'EL PALOMAR, Argentina', 'Ciudad de Buenos Aires, Arg', 'Capital Federal', 'Cap. Federal / Almagro', 'Buenos Aires - ARGENTINA', 'Saavedra, Capital Federal', 'Microcentro, CABA, Argentina', 'Gorriti 4735 Palermo Soho', 'Buenos Aires - Ramos Mejia', 'Balvanera', 'Moron', 'Hurlingham,Buenos Aires', 'en Bs. As.', 'La Tablada, Buenos Aires', 'Bueno Aires', 'buenos aires,argentina', 'Ciudad Autónoma de BuenosAires', 'Villa Ballester, bs as', 'San Andrés Buenos Aires', 'Quilmes - Argentina', 'Buenos Aires Town', 'Banfield, Buenos Aires.', 'avellaneda', 'San martin', 'Quilmes Oeste,Bs As Argentina', 'Ituzaingó, Argentina', 'Ituzaingo, Argentina', 'Boedo, Argentina', 'Boedo, Buenos Aires', 'Bella Vista & Caseros', 'lomas del mirador', 'iPhone: -34.562439,-58.466797', 'Villa Urquiza', 'San fernando', 'caseros', 'Villa Gral Mitre, CABA', 'De Urdi. Entre Ríos.Bs. As-Arg', 'Almagro,Capital Federal', 'Tigre, Bs. As.', 'Palermo Out, CABA, Argentina', 'Boedo - Argentina', 'Quilmes, Buenos Aires', 'La Plata,Bs As,Argentina', 'Banfield Argentina', 'haedo', 'Villa del Parque, Comuna11', 'Donde no me jodan. Cap. Fed.', 'Coronel Dorrego Buenos Aires', 'Ramos Mejia', 'La Plata. Argentina', 'Ciudad Magica de Buenos Aires', 'BANFIELD', 'Palermo, Capital Fedetal', 'Avellaneda - Buenos Aires', 'Villa Ballester, Buenos Aires.', 'Quilmes Bs.As Argentina', 'Gran Buenos Aires', 'Caseros. Buenos Aires.', 'Argentina, Bs As, Martínez', 'Almirante Brown', 'almirante brown', 'San Miguel de Tucumán', 'San Martín', 'Palermo- Argentina', '-34.438311,-58.749588', 'pilar', 'argentina buenos aires', 'URUGUAY 153,Morón, Argentina', 'Ranelagh', 'Ramos Mejia - Buenos Aires', 'Lomas de Zamora Buenos Aires.', 'Lanus Oeste', 'Haedo', 'Gurruchaga 1519, C.A.B.A.', 'Floresta-Bs As-Argentina', 'Recoleta.CABA', 'Lanús este, Argentina', 'La Plata; Pcia Bs.As, Arg.', 'Hurlingham, Buenos Aires', 'Escobar, bs as.', 'Buenos Aires • Argentina', 'Buenos Aires / Santo Domingo', 'Bella Vista, Buenos Aires', 'Argentina ,Buenos Aires', 'Recoleta, Buenos Aires', 'Caballito, BAires, Argentina', 'Melmac Colegiales', 'General Rodriguez', 'lanus-argentina', 'San fernando, buenos aires', 'Nuñez, Buenos Aires', 'Lomas De Zamora', 'Buenos Aires! ... Argentina =D', 'boedo']  **Reference (Foreign) Locations** = ['Venezuela', 'Cali Valle del Cauca', 'Santiago, Chile', 'Caracas, Venezuela', 'Punta Arenas, Chile', 'Montevideo, Uruguay', 'Bogotá, D.C., Colombia', 'Colonia, Uruguay', 'La gran caracas.', 'Chile', 'Orlando, FL', 'Paraguay', 'Colombia', 'Laviana', 'Brasil', 'Uruguay', 'Armação dos Búzios, Brasil', 'Colonia - Uruguay', 'México', 'Lima, Peru', 'Caracas', 'España', 'São Paulo, Brasil', 'Florida', 'valencia,venezuela', 'Santiago de Chile', 'Sao Paulo, Brasil', 'Caracas-Venezuela', 'Argentina / Colombia', 'Asuncion, Paraguay', 'Maracay, Venezuela', 'Barranquilla/colombia', 'Dubai, EAU', 'New York, NY', 'ÜT: 10.475898,-66.896853', 'California, USA', 'Asunción, Paraguay', 'Termas de Río Hondo- Buenos Ai', 'Bolivia', '🇮🇹', 'Barcelona, España', 'Bogota', 'Versailles', 'Boulogne', 'Madrid', 'Costa Rica', 'Brazil', 'Paraguay 2590 Recoleta Bs As', 'Bogotá, Colombia', 'Miami Beach, FL', 'New York City', 'Los Angeles, CA', 'Rio de Janeiro, Brasil', 'Distrito Federal, Brasil', 'United Kingdom', 'Latam - USA', 'Miami, FL', 'Lima, Perú', 'URUGUAY', 'Mexico', 'caracas', 'Trujillo - Venezuela', 'Bogotá', 'Perú', 'Dublín, Irlanda', 'Valencia, España', 'Argentina Londres', 'venezuela', 'New Zealand', 'New York', 'Escobar', 'Nueva York, USA', 'lima', 'París, Francia', 'Caracas - Venezuela', 'Quantico', 'Barquisimeto, Venezuela', 'Mexico City', 'Estados Unidos', 'Ciudad de México', 'montevideo 1480 TE 48113701'] |
