# Supplementary material for: Geolocation of multiple sociolinguistic markers in Buenos Aires
Source: PLoS One. 2022 Sep 9;17(9):e0274114. doi: 10.1371/journal.pone.0274114 (PMC9462814; doi:10.1371/journal.pone.0274114)
Supplement: S5 File — (DOCX) [file pone.0274114.s005.docx]

Supplementary File S5

Differential distributions and frequency-comparison plots for cases with large asymmetry in tweet vs reference count numbers. These figures show that the patterns in the differential-distributions and the shape of the frequency-comparison plots are not strongly affected by the count asymmetry. The statistics in the supplementary information (S4 File) also confirm an insensitivity to the count asymmetry.


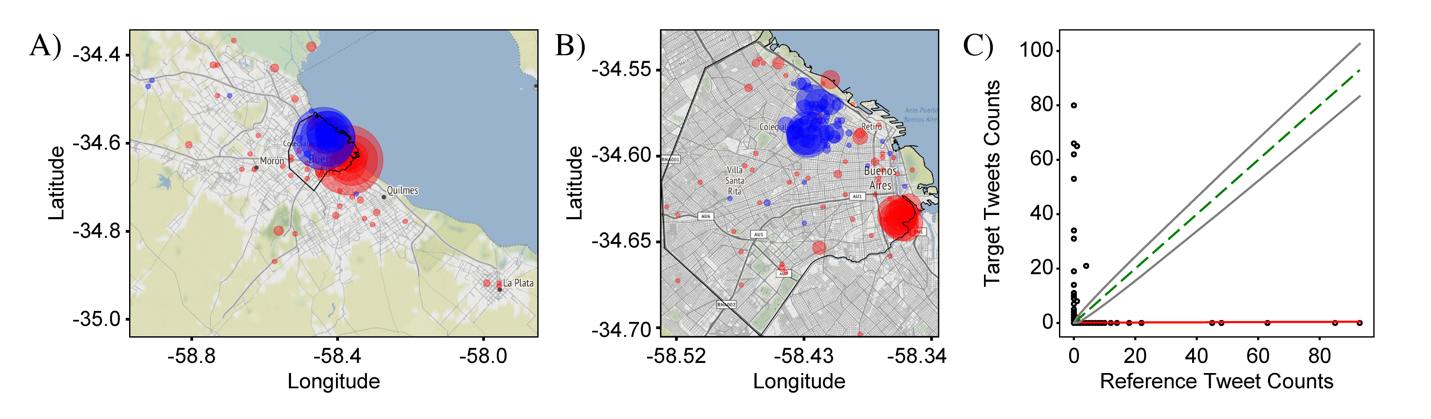


S5 Fig 1. Neighborhood Names case, with equalized counts. Base map and data from OpenStreetMap and OpenStreetMap Foundation under the Open Database License (S5 Fig 1A,B).


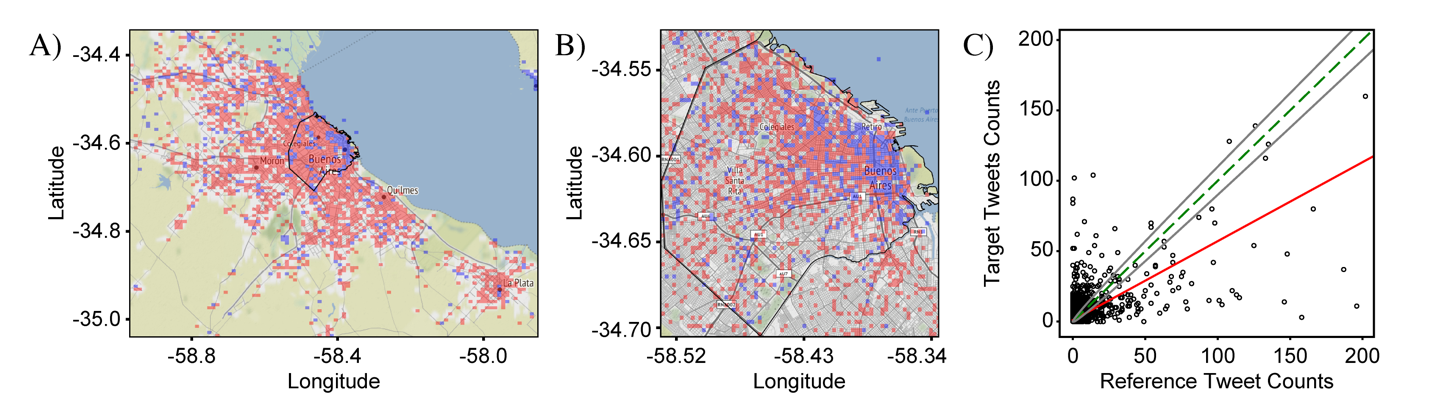


S5 Fig 2. User Origin case, with equalized counts. Base map and data from OpenStreetMap and OpenStreetMap Foundation under the Open Database License (S5 Fig 2A,B).


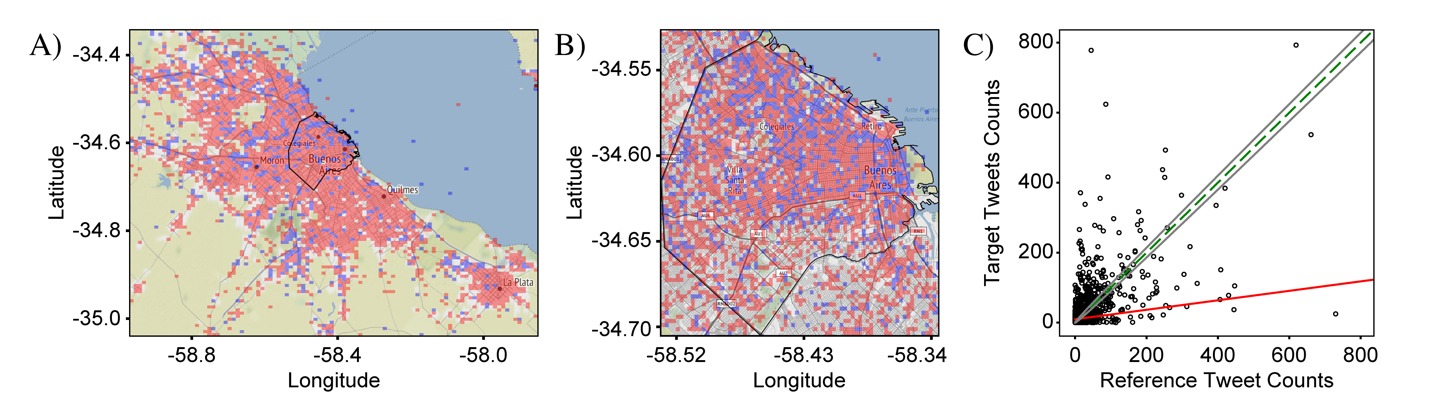


S5 Fig 3. Tweet Language case, with equalized counts. Base map and data from OpenStreetMap and OpenStreetMap Foundation under the Open Database License (S5 Fig 3A,B).
